# Evaluations of the Disease Surveillance Centre network in Scotland: What parts has it reached?

**DOI:** 10.3389/fvets.2023.1099057

**Published:** 2023-02-21

**Authors:** Andrew J. Duncan, Jude I. Eze, Franz Brülisauer, Julie M. Stirling, Amy Jennings, Sue C. Tongue

**Affiliations:** ^1^Centre for Epidemiology and Planetary Health, Department of Veterinary and Animal Science, Northern Faculty, Scotland's Rural College (SRUC), Inverness, United Kingdom; ^2^UHI Inverness, University of the Highlands and Islands, Inverness, United Kingdom; ^3^Biomathematics and Statistics Scotland, Edinburgh, United Kingdom; ^4^SRUC Veterinary Services, Inverness, United Kingdom; ^5^The Royal (Dick) School of Veterinary Studies and The Roslin Institute, University of Edinburgh, Edinburgh, United Kingdom

**Keywords:** disease surveillance, evaluation, network, veterinary, farmer, passive surveillance, livestock

## Abstract

Regular evaluation is a prerequisite for systems that provide surveillance of animal populations. Scotland's Rural College Veterinary Services' Disease Surveillance Centre (DSC) network plays an integral part in surveillance to detect new and re-emerging threats within animal populations, predominantly livestock. In response to surveillance reviews and proposed changes to the network, an initial evaluation of diagnostic submissions data in 2010 to mid-2012 established a baseline “footprint,” while highlighting challenges with the data. In this recent evaluation for the period 2013–2018, we developed a new denominator using a combination of agricultural census and movement data, to identify relevant holdings more accurately. Iterative discussions between those processing submissions data and those involved in collection at source took place to understand the intricacies of the data, establish the most appropriate dataset, and develop the processes required to optimise the data extraction and cleansing. The subsequent descriptive analysis identifies the number of diagnostic submissions, the number of unique holdings making submissions to the network and shows that both the surrounding geographic region of, and maximum distance to the closest DSC vary greatly between centres. Analysis of those submissions classed as farm animal post-mortems also highlights the effect of distance to the closest DSC. Whether specific differences between the time periods are due to changes in the behavior of the submitting holdings or the data extraction and cleaning processes was difficult to disentangle. However, with the improved techniques producing better data to work with, a new baseline footprint for the network has been created. This provides information that can help policy makers and surveillance providers make decisions about service provision and evaluate the impact of future changes. Additionally, the outputs of these analyses can provide feedback to those employed in the service, providing evidence of what they are achieving and why changes to data collection processes and ways of working are being made. In a different setting, other data will be available and different challenges may arise. However, the fundamental principles highlighted in these evaluations and the solutions developed should be of interest to any surveillance providers generating similar diagnostic data.

## Introduction

Systems that provide surveillance of animal populations can be implemented to meet either one, or more, specified objectives. Examples of such objectives could be to: allow declaration of freedom a specific disease; determine the frequency of a disease in a population or, detect a new or emerging threat.^1^ Priorities for surveillance systems should be identified when the infrastructure is being built or reformed, and the success of them should be measured against set criteria. However, it is recognised that both priorities and success criteria will be subject to iterative adaptation and evolution to meet changing needs of those commissioning the system, be they industry, government state, or other stakeholders. Regular review, monitoring and evaluation is required. Such reviews should provide information about whether the surveillance system is generatits intended outputs and meeting objectives, whether these objectives appropriate at the current time and for the visible future, and therefore whether impronts or changes are required (1, 2). Evaluation of the existing system can also provide a baseline against which to measure the ef of proposed change. These reviews are, therefore, an essential step in the policy cycle (3).

A systematic review in 2015 (4), identified three evaluation approaches available in the field of animal health surveillance (5–7). These approaches provide either a general or a structured approach (6), methods (7), or a tool (5) for the evaluation process. More recently, in 2019, an additional tool for integrated evaluation has been developed, tested, and demonstrated (2). These tools and approaches are often time-consuming and resource intensive to apply in full. The idea that evaluations should be individually tailored was highlighted for public health systems by Klaucke (8), and has been recognised in the United Kingdom's approach to animal health surveillance.^1^ The expectation is that each of the four administrations (England, Wales, Scotland, and Northern Ireland) will independently evaluate the performance of their own animal health surveillance systems.

Up until 2019 Scotland's Rural College (SRUC) Veterinary Services (VS) provided a network of eight Disease Surveillance Centres (DSCs) in Scotland to support livestock disease surveillance through submissions of vet and farmer-selected samples and carcasses that were submitted for diagnostic purposes and post-mortem (PM) examination. In 2019, the PM room capacity of this network was reduced,^2^ although the services of the network continue to be delivered by SRUC VS, on behalf of the Scottish Government.

The submissions assist with improving animal health at the farm level, while the diagnostic information is available, and contributes to, the passive surveillance system (9, 10) both in Scotland and across Great Britain (GB).^3^,^4^ Comparable networks exist within^5^ and across other countries.^6^ These networks, when enhanced and developed, have been shown to be an increasingly viable method to observe new patterns in endemic diseases and to identify new diseases (11–13).

A fundamental requirement for the effectiveness and efficiency of such networks is engagement and participation. Previous studies have shown the importance of the individual in surveillance (10, 14, 15). Whether it is the farmers themselves (11) or the veterinarians working with those farmers (16–18), these individuals act as gate-keepers and can have an impact on when and how disease is reported. An understanding of how the network is used can help policy makers determine if access to the network is appropriate and if it is providing sufficient coverage to allow conclusions to be drawn with confidence. The first step in an investigation of drivers for submission is to look at how the network is used and whether there are links between surveillance submissions and geographic location (19), or distance to a laboratory (20).^7^

Between 2011 and 2019, the British network underwent significant review and restructure.^6^

As part of background evidence for policy advisors, the organisational attributes of coverage and usage from 2010 to mid-2012 of the Scottish DSC network, by Scottish livestock holdings with any of the main target species (cattle, sheep and pigs), was evaluated.^8^ However, it was challenging to provide complete analysis of the network usage and drivers of that usage because of systematic issues in the data collection that were identified.

In conjunction with Scottish Government science and policy advisors, it was decided that it would be appropriate to re-evaluate the SRUC VS diagnostic submissions data for the period 2013–2018. The primary aims were to evaluate if the initial quality, and thus utility, of data had improved therefore providing more reliable and complete geolocation data on submitting holdings, to determine coverage and usage for the new study period and to investigate the spatial pattern of usage of the DSC network by these livestock holdings. If these aims could be achieved, the outcome would be the provision of a baseline against which the restructured network could be re-evaluated and an assessment of whether the data are suitable for further analysis of factors that drive network usage. Here we present the evaluation process and results for 2013–2018; we compare the outputs to those from the 2010 to mid-2012 evaluation and provide discussion on any differences identified.

## Materials and methods

### The Disease Surveillance Centre network

Prior to 2019, there existed a network of eight DSCs located across Scotland. These were facilities, run by SRUC VS, for PM examination, as well as for diagnostic testing. The range of diagnostic tests run on-site differed between DSCs, but all included parasitology and bacteriology. If required, samples could be forwarded into the network for additional diagnostic testing that was not available on-site, such as serological and molecular testing as well as histopathology and biochemistry.

Submissions, each relating to a single animal or group of animals from a single holding, were made to a DSC, often by the consulting vet. These submissions, or samples from them (for example faeces, blood, tissue samples), could either be dealt with directly or passed on internally to another DSC within the network. The submissions included in this analysis were labelled as having been submitted for diagnostic purposes, rather than monitoring of healthy animals. A subset were identified as postmortem farm animal (PMFA).

### Holdings dataset – the denominator

Before we were able to analyse how the DSC network was used, it was crucial to establish the type and location of any holdings who might potentially make a submission. The aim with the denominator dataset was to identify all holdings in Scotland that had at least one animal of any of the major livestock sector species i.e., bovine, ovine, or porcine. These are the by far the most common species submitted to the surveillance network.

There is a statutory requirement that anyone having at least one of the animals in this species list on their property is required to be registered as a “holding”.

Within GB, the term “holding”, when applied to livestock, usually describes the land and buildings that people use for keeping livestock,^9^ including livestock kept as pets. Each holding is assigned a unique county parish holding (CPH) number. CPH numbers have the format 12/345/6789, where the first two digits represent the county, the next three the parish and the final four an individual holding within the parish. A CPH number can be temporary, or permanent and it can cover a range of land and buildings within a specified distance from a main livestock handling area. A livestock business may, however, consist of have more than one CPH. The CPH number and the term “holding” therefore approximates to, and usually is, the basic unique identification of a farm used in many British livestock recording systems. However, care is needed when handling datasets as other types of premises and/or land such as markets, lair ages, slaughterhouses, ports and showgrounds have CPH numbers. A CPH is also necessary to comply with the legislative requirements for recording and reporting of livestock movements.

To identify holdings of interest information from three separate sources were combined: the agricultural census; the Cattle Tracing System (CTS); and the ScotEID database. The agricultural census takes place every June and collates, amongst other details, the number of cattle, sheep and pigs on each livestock holding.^10^ The CTS database is used to record all births, movements onto and off holdings, and deaths of cattle within the UK. It is managed by British Cattle Management Service (BCMS)^11^ and access to these data is granted by the Animal and Plant Health Agency (APHA).^12^ ScotEID^13^ is the livestock traceability system for Scotland managed by the Scottish Agricultural Organisation Society on behalf of the Scottish Government. It was used for sheep and pig movements records in the study period, although from 2022 ScotEID it also holds the cattle tracing system. Any sheep and pig movements that contain all or part of their movement within Scotland are recorded in ScotEID. The study period was 2013–2018. All the datasets used in this study were provided to the Scottish Government's Centre of Expertise on Animal Disease Outbreaks (EPIC) and stored in the EPIC data repository, which is a centrally curated collection of data resources.

The agricultural census and movement data were extracted and cleaned separately for each species. The sheep data includes information on batch movements and individual animals, whilst the cattle data is stored solely as individual animal movements. Pig movement data is only recorded in batches. Holdings were identified as having sheep or pigs if they had animals move off their holding at any point in the study period, as by proxy they then must have had animals on that holding, which may have needed diagnostic services. To ensure all cattle holdings were identified, these were included if they had movements onto their location, as each individual cattle birth is recorded as a movement onto the holding but with no “off location” (21). Some small herds could have no movements off within a year, with the only change being births.

For all species, every attempt was made to restrict the data to locations identified as agricultural holdings, rather than another type (e.g., market or abattoir). Inclusion in the agricultural census automatically identified a location, otherwise it was dependent on species. In the cattle data, the “premises type” identifier was used and only those marked as agricultural holding were retained. For the sheep and pig data, only holdings identified as livestock units were included in the final holdings denominator dataset.

A holding was identified as having an individual species based on having a non-zero value in the agricultural census or if recorded as moving that species of animal between 2013 and 2018. The holdings identified for each species were then amalgamated to provide a dataset to represent all Scottish holdings.

### Submissions records – the numerator

As with the process of identifying the holdings that could use the DSC network, it was necessary to combine multiple datasets to establish which holdings had made submissions. The first step in the process was to extract submission records from all eight DSCs. At the time of the extraction, the eight DSCs operated eight independent Laboratory Information Management Systems (LIMS). The LIMS had the same structure and interacted with each other. Each submission was given a unique identifier that was held across all samples and any internal submissions generated. Some of the tables in the individual LIMS were the same but, crucially, the clients table was different for every DSC. This meant that the same holding could be included in the clients table of multiple DSCs. Furthermore, client tables allowed multiple entries of the same farm within a particular LIMS.

Individual holdings were identified in LIMS by their CPH number. The CPH was sometimes missing from submissions and in other cases the CPH number recorded was not in the correct format. Efforts were made to both clean the data (for example correcting CPH form (e.g., 123/456/789 corrected to 12/345/6789) and to match submissions to previous submissions with the same address to allow identification of the CPH (for example by using another unique identifier of a concatenation of the name and postcode on the submission). There were, however, 134 farms where the submissions had the same name and postcode but different CPHs recorded. In these cases, the CPH number in the earliest submission was used.

The submitting holdings were linked to the eligible holdings using the CPH and a single Easting and Northing was recorded with that CPH. At this point, any holdings that were out with Scotland were removed from the data set.

Finally, the straight-line distance of all the holdings to each of the DSCs was calculated. This enabled the identification of the geographically closest CPH for each holding. In turn this allowed catchment areas to be created for every DSC i.e., the group of holdings where a particular DSC was the closest centre (or entry point to the network) for each of them.

### Descriptive analysis using both datasets

Having completed construction of both submission and holding datasets, the geographical distribution of all Scottish livestock holdings was visualised, as was the distribution for each individual species. The holding and submission datasets were also combined to construct density ratio maps (kernel density 10 km^2^, grid cell size 1 km^2^) to examine the proportion of all Scottish livestock holdings that submitted from a specific area.

The submission data from 2013 to 2018 were described in terms of the numbers and proportions (or percentage) of holdings submitting (a) to the network, (b) to their closest centre and (c) according to their distance from their closest centre. PMFA submissions were described separately as well as in conjunction with all diagnostic submissions. The descriptive outputs for this 2013–2018 evaluation were then placed in the context of those from the 2010 to mid-2012 period.

All descriptive spatial analysis was carried out using qGIS (22) with all other results calculated in R (23), using the diverse suite of packages (24) and plots created using ggplot2 (25).

## Results

### Holdings dataset – the denominator

Having extracted and cleaned the holding dataset as described above, 24,057 livestock holdings were identified across Scotland in the period 2013–2018 inclusive (Table 1). The total number of pig holdings is of the order of a tenth of those with cattle and a sixteenth of those with sheep. It should be noted that a holding is counted identically regardless of the number of animals present, so a smallholding with one cow, sheep or pig is treated the same as a holding with 300 animals.

**Table 1:** The number of Scottish livestock holdings by species in the period 2013 - 2018 inclusive, as derived from demographic and movement data.

**Species on holding**		Number of holdings with > 0 animals^*^	Per cent (%) of all Scottish livestock holdings^*^
Total livestock holdings		24,057	100%
	Bovine/cattle	12,513	52.0
Livestock holdings with named species present	Ovine/sheep	19,374	80.5
	Porcine/pigs	1,327	5.52

^*^Holdings appear more than once if they were recorded as having more than one species.

**Figure 1:**
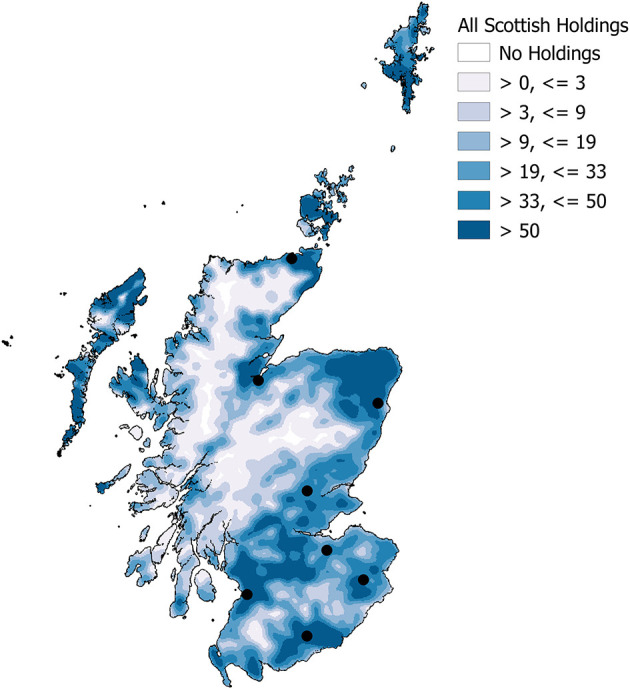
All Scottish livestock holdings 2013 - 2018 inclusive, as defined from census and movement data - Kernel density 10 km radius, number of holdings per 10 km-square - with the locations of the eight DSCs (black spots).

The spatial distribution of livestock holdings across Scotland was heterogeneous (Figure 1). The eight DSCs (marked as black dots in Figure 1) were located in livestock-holding dense areas The higher densities for all livestock holdings observed in the South of Scotland, Orkney and Shetland were due mainly to cattle (Supplementary Figure SM1) and sheep (Supplementary Figure SM2) holdings. The majority of pig holdings were located in the North-East (Supplementary Figure SM3).

**Figure 2:**
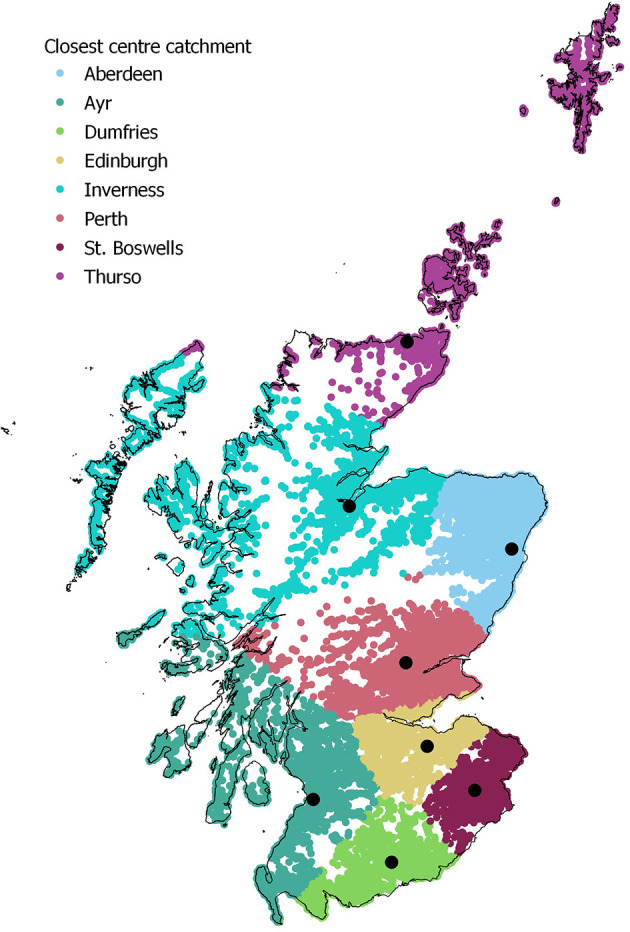
All holdings within the denominator dataset coloured by their closest DSC, which represents their closest point of entry to the network.

The spatial distribution of holdings was not uniform across the DSC network when they were classified by their geographically closest DSC (Figure 2). The DSCs in the South of Scotland had a smaller geographical catchment area when compared to the two most northerly centres, Inverness and Thurso (furthest North).

The numerical distribution, like the spatial, was not uniform across the network, with the most northerly regions having the highest number of individual holdings (Table 2). The numbers of holdings for each DSC were consistently slightly higher in 2013–2018, compared to the corresponding values derived from the different denominator used for 2010 to mid-2012. There were slight shifts in the percentage frequency distribution (Table 2).

**Table 2:** The number and percent of all the Scottish livestock holdings that have the stated DSC as their closest centre, i.e. were within its catchment area, for 2013 – 2018 and (2010 to mid-2012).

**DSC name**	**Scottish livestock holdings for which a specific DSC is the “closest centre”**	
		**Number of livestock holdings 2013 - 2018 (2010 to mid-2012)**	**% of all Scottish livestock holdings 2013 - 2018 (2010 to mid-2012)**
Inverness	6,480 (5,563)	26.9 (25.5)
Thurso	3,520 (3,315)	14.6 (15.2)
Ayr	3,429 (3,241)	14.3 (14.9)
Aberdeen	3,415 (3,203)	14.2 (14.7)
Perth	2,355 (2,112)	9.8 (9.7)
Dumfries	2,057 (1,841)	8.6 (8.5)
Edinburgh	1,665 (1,545)	6.9 (7.1)
St Boswells	1,136 (964)	4.7 (4.4)
Total	24,057 (21,784)	100 (100)

**Figure 3:**
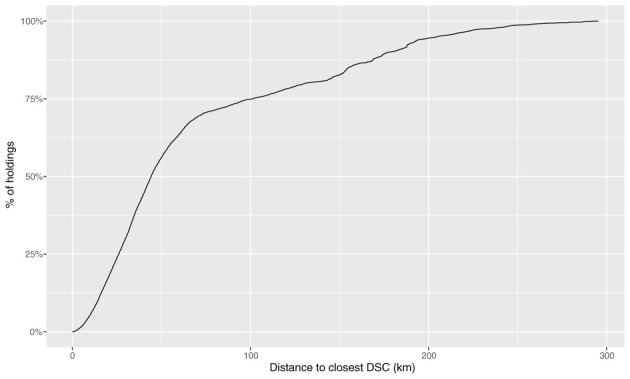
The cumulative frequency (%) of Scottish livestock holdings (2013 - 2018) by distance (km) from the existing closest network entry point (closest existing DSC) for each livestock holding.

Across all centres, just over half (56.1%) of the holdings were within 50 km (straight line distance) of their closest centre (Figure 3). This was very similar to 2010 to mid-2012 where it was ~57% of all such holdings. Cumulative distribution plots for each centre show that the percentage of holdings located within 50 km varies widely between DSCs (Supplementary Figures SM4–11). Five of the eight DSCs had at least 75% of holdings within 50 km (Supplementary Figures SM4, 6, 7, 9, 10) and the two most northerly DSCs both had <40% of their closest holdings within 50 km (Supplementary Figures SM8, 11).

### Submission records – the numerator

In the 2013–2018 evaluation there were 86,996 diagnostic submission records initially extracted, of which 67,360 records had CPH numbers recorded in the correct format. For seven of the eight DSCs this represented between 83 and 90% of their submission records. However, the overall network figure is 77.4% because of one DSC for which more than half of its records did not have valid CPH numbers. This represented a substantial improvement from the first evaluation (2010 to mid-2012), where all DSCs initially had at, or below, 70% of records with CPH in the correct format and three had <50%. In the current evaluation, after all cleaning was completed, the proportion of valid CPHs for the overall network had risen to 80.1%. This was mainly due to improvements in six DSCs (now with a range of 84.6–90.1%).

Just over one in four (26.3%) Scottish livestock holdings made at least one diagnostic submission of any type, to the network, in 2013–2018. This is a similar, but slightly higher estimate than that obtained from 2010 to mid-2012 (23.4%).

At the end of the submission records extraction and cleaning process, 40,564 individual diagnostic submissions were identified as having been submitted from at least 6,322 unique Scottish livestock holdings, during 2013–2018. This compares to 34,035 submissions from 5,095 unique Scottish holdings identified in the first evaluation (2010 to mid-2012).

**Figure 4:**
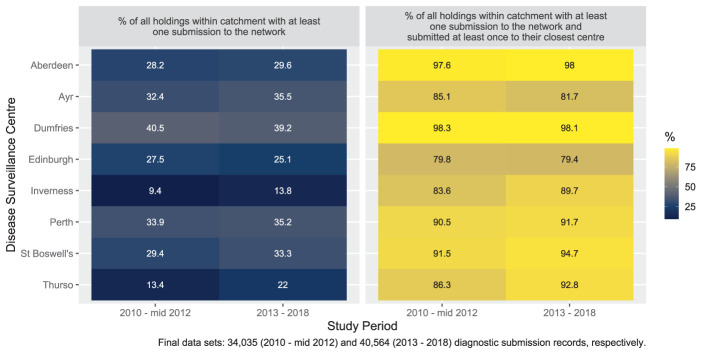
Distributions (%) of holdings by named DSC catchment area - aspects relating to all types of diagnostic submissions for 2010 to mid-2012 and 2013 - 2018.

Figure 4 shows the percentage of Scottish livestock holdings making a diagnostic submission to the network and those who made a submission and submitted to their closest DSC. The results are split into the catchment areas of the individual DSCs. The results from 2013 to 2018 are comparable with the earlier results from 2010 to mid-2012 with submission rates varying between the individual DSCs. Submitting livestock holdings in the Edinburgh and Ayr catchment areas were least likely to have made at least one diagnostic submission to their closest DSC in 2013–2018, whilst Aberdeen and Dumfries catchment area holdings were most likely to submit to their closest DSC. Across the entire network 10% of holdings who submitted, did not make any diagnostic submissions to their closest DSC (Supplementary Table SM1). There were some apparent changes between the studies. The percentage of livestock holdings submitting at least once to the network was higher in six of eight DSC areas. The percentage of livestock holdings in a named catchment area that submitted at least once to the network and submitted at least once to their closest centre was lower in one centre (Ayr). This value was more than 2% higher in three DSCs, with the largest increases observed for the two most northerly centres, Inverness and Thurso (Figure 4).

Of the 40,564 diagnostic submissions from 2013–2018, there were 8,342 classed as PMFA. During this period, more than one in ten holdings (12.2%) made at least one PMFA submission, to the DSC network. This is a higher estimate than the 9.4% observed in 2010 to mid-2012.

**Figure 5:**
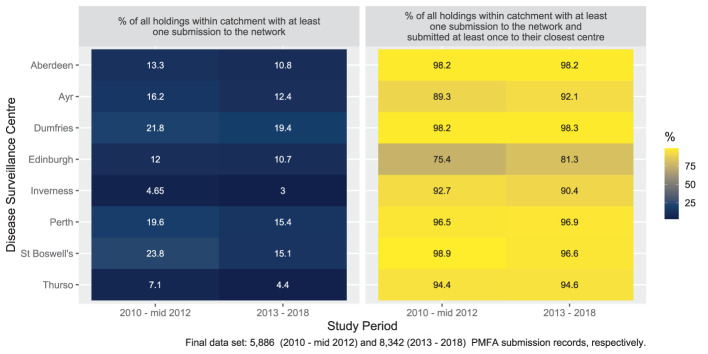
Distributions (%) of holdings by named DSC catchment area - aspects relating to PMFA submissions for 2013 - 2018 and 2010 to mid-2012.

Figure 5 shows the percentage of Scottish livestock holdings making a PMFA submission to the network and those who both made a submission and submitted to their closest DSC. The results are split into the catchment areas of the individual DSCs. The complete numerical values are shown in Supplementary Table SM2.

**Figure 6:**
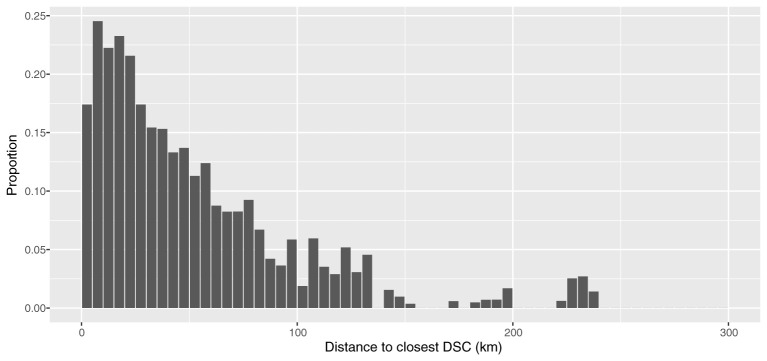
Proportion of all Scottish holdings making at least one PMFA submission, during 2013 – 2018, by the distance (in 5 km groups) to their closest DSC.

Of the holdings that made PMFA submissions to the DSC network in 2013–2018, just over one out of 20 did not make one to their closest centre (6.1%, Supplementary Table SM2). This is slightly higher than was observed in 2010 to mid-2012 and appears to be predominantly due to apparent changes among Edinburgh catchment area PMFA submitting holdings. A lower percentage of these holdings submitted their PMFAs to Edinburgh (their closest centre) in 2013–2018 (75.4%) than they did in 2010 to mid-2012 (81.3%). This was the only centre where the percentage of holdings submitting PMFA to their closest centre, given they made a PMFA submission at all, was lower when compared to all diagnostic submissions (Figure 4). The proportion of all Scottish holdings that made at least one PMFA submission to the DSC network is negatively correlated with distance to their closest centre (Figure 6). A similar, but weaker relationship, was found when all diagnostic submissions were examined (Supplementary Figure SM12).

For all diagnostic submissions (not just PMFAs), most holdings submitted only to their closest DSC. However, there were 547 holdings that did not make any submissions to their closest DSC (Supplementary Figure SM13). These holdings were well distributed throughout the locales of the individual DSCs and are not restricted to a particular catchment area (Supplementary Table SM3).

### Descriptive spatial analysis of network usage

**Figure 7:**
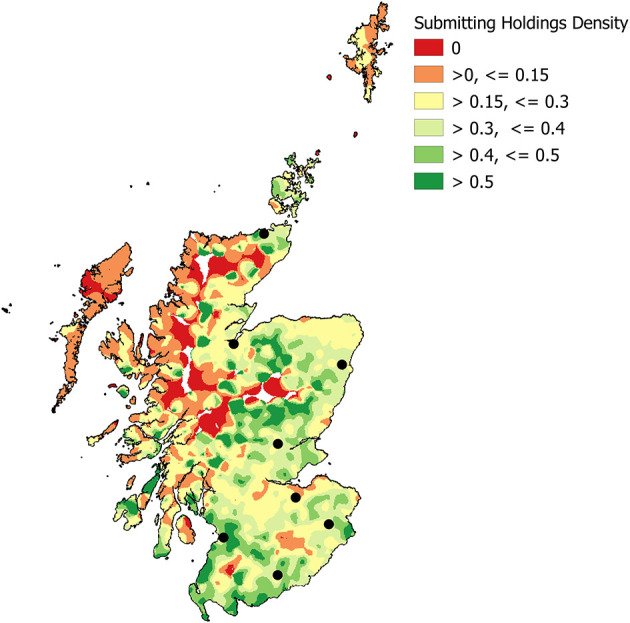
Density ratio – the proportion of Scottish livestock holdings who made at least one diagnostic submission to the network in the period 2013 - 2018 (Kernel density, 10 km radius, grid cell size 1 km^2^) with the locations of the eight DSCs (black spots).

There were a few areas where livestock holdings were present but <15% made at least one diagnostic submission of any type to the DSC network in the period 2013–2018 (Figure 7). These areas were mainly along the North West coast and in the Western Isles, although such areas were scattered across other islands and remote areas, as well as a few more southern and eastern areas. Areas where more than half of the livestock holdings have made one or more submissions in the time period were not necessarily those closest to the DSCs.

**Figure 8:**
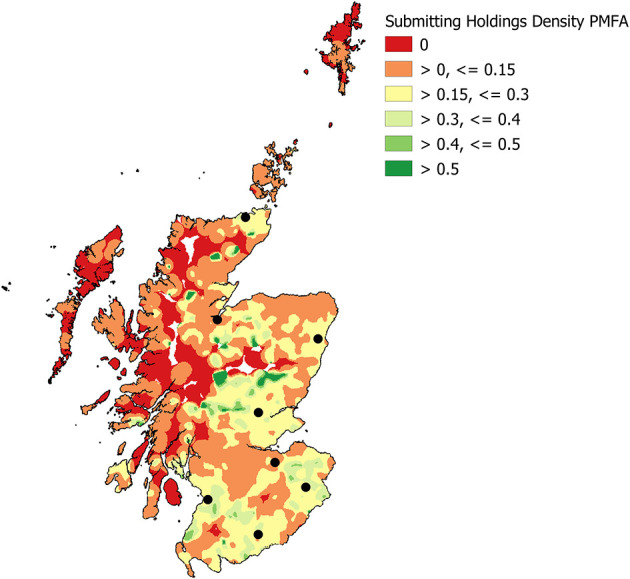
Density ratio – the proportion of Scottish livestock holdings who made at least one post-mortem farm animal (PMFA) diagnostic submission to the network in the period 2013 - 2018 (Kernel density 10 km radius, grid cell size 1 km^2^) with the locations of the eight DSCs (black spots).

The under-represented areas became larger when the dataset was limited to the subset of livestock holdings that made at least one PMFA submission (Figure 8). The reduction in those areas where more than half of the holdings have submitted was also noticeable.

## Discussion

We have evaluated the potential coverage and the usage of the Scottish network of disease surveillance centres for the period 2013–18 and estimated how far it reached i.e., we have described the footprint of submitting holdings during this period. During the evaluation, we developed a comprehensive extraction and cleaning process for the submission records; highlighted areas for consideration for improvement in the data collection process; developed a process for extracting a comprehensive denominator dataset for Scottish livestock holdings from existing demographic and movement datasets, and improved our confidence in the outputs compared to the earlier evaluation. This has enabled us to a produce a robust assessment of the performance of the DSC network, in terms of the attributes of coverage and data quality. It has facilitated the production of information about usage and the relationship with distance and established a baseline reference for both the surveillance provider and science-policy advisors.

With improved confidence in the data and analytical outputs, compared to the 2010 to mid-2012 evaluation, we can now start to propose potential hypotheses, with regards to drivers for submission and to propose areas that need considering, if improvements to data quality, or usage of the network, are required.

### Comparison with previous study

While it is tempting to compare the results from the analysis of submissions from 2013 to 2018 with those from 2010 to mid-2012, this should be done with extreme care; several difficulties arise. Both the submission and holding datasets - numerator and denominators - in this re-evaluation were created using some of the lessons learned from the initial one. This time, with the longer period analysed, the holding dataset made use of movement data in addition to the agricultural census used earlier. The data from the census represents a snapshot of the animals on Scottish holdings. Using only a single snapshot of a single year is unlikely to produce an accurate picture of the holdings that could potentially make a submission. For example, due to their mobility and the sheep calendar year effect, the Animal and Plant Health Agency's Livestock Demographic Data Group^14^ prefer to use the end of year Sheep and Goat Inventory data for sheep. This dataset was not available to us. Any change identified in the denominator could be due to an actual change in the industry sectors^15^ over time, a change due to the methods, or both.

Similarly, the extraction of the submission dataset and particularly the cleaning/matching of CPH numbers went through an, in our opinion, improved process. This was due to our ability, for this re-evaluation, to bridge the gap between those collecting and managing the data and those using it. SRUC VS personnel were integrated into the evaluation team from the outset. They were able to explain some of the idiosyncrasies of the LIMS used by the DSCs and co-construct approaches, as outlined in the methods section. One outcome of the re-evaluation was an improved understanding of how submitted samples are recorded at receiving DSCs, how they are recorded if they are subsequently moved within the network for the purposes of diagnostic testing and how these processes relate to submission records. As these processes were considered in the new data cleaning protocol, we can be more confident that we have not over-estimated the effect of incorrect CPHs and double-counted samples. Any apparent change in the numerator dataset when comparing 2013–2018 and 2010 to mid-2012, could be due to several factors. These include the cleaning process, changes in propensity to submit, an increase in disease events over the period, increased days at risk, or a mixture of any of these factors and so should be interpreted with care.

The results of this re-evaluation suggest that accurate recording of CPH numbers has improved in the data from 2013 to 2018 compared with 2010 to mid-2012. The original evaluation and its outcomes may have contributed to this overall improvement in accurate CPH recording, as it did include substantial hand-cleaning of the 2010 to mid-2012 submissions data by DSC staff. Another possible contributing factor is improved completion of CPH on submission forms by submitting private veterinary practitioners. This may be an effect of the Scottish Government's Bovine Viral Diarrhea eradication scheme.^16^ Launched in 2012, CPH numbers were, and still are, mandatory for submissions made for this scheme, potentially leading to increased familiarity and compliance with completion of CPH numbers requested on other submission forms.

However, there was one DSC for which the new process could still not achieve as good an endpoint, in terms of the percentage of CPHs in the correct form, as in the original evaluation. This is most likely due to the receipt at this DSC of substantial numbers of submissions from holdings across Scotland and non-Scottish holdings, whereas other DSCs tend to receive submissions predominantly from the local area. On receipt of samples at these DSCs, staff are more likely to be able to use local knowledge to supplement sparsely completed submission forms. This will lead to a greater proportion of CPHs that are entered in a valid format. Similarly, it is likely that, in the original re-evaluation process, the hand-cleaning process was able to identify and delete inappropriate submission records in a way that cannot be matched by rule-based algorithms and methods. This could have led to an apparent improvement in the performance at this specific DSC. It is also possible that the phased implementation of changes to the network during the latter part of the evaluation period, and increased centralisation of diagnostic testing may have resulted in more submissions entering the network *via* a different DSC. At the entry-point DSC, the CPH could have been recorded accurately; it would not necessarily be recorded on receipt internally at the final DSC. The new cleaning process should, however, have captured these records.

Despite the improvement in correctly recorded CPHs and given all the caveats stated above about making inter-evaluation comparisons, the overall results from the two evaluations are remarkably similar. This provides some additional confidence that these missing data do not have a major effect on the answers to the questions being asked here. In an ideal world there would be little to no incomplete, or missing data. However, a balance must be found that enables operations to be conducted within the resources available, while optimising the utility of the data collected. One possible way of addressing this correct CPH issue would be for an automated cross-check between the data entered and a regularly updated master list of the CPH register. The latter is not available to the DSC network for the purpose of routine diagnostic submissions. Another option considered in the past was to provide a discount to clients where a valid CPH is provided on the submission form.

### Submissions

Individual farmer or veterinarian preference over which DSC to utilise can be influenced by professional relationships (10, 28) with the SRUC VS personnel. This may be based on perceived knowledge and experience, be it local, disease or species-specific expertise and may apply particularly when the submission can be delivered by a third party e.g., posted. Location of laboratories and quality of advice were the two key features identified in a questionnaire survey, which informed the 2011 Review of Veterinary Surveillance (see text footnote 7). Although we have not explored these aspects in the current evaluation, there was some evidence of a species expertise effect associated with porcine samples in the earlier evaluation (data not shown).

As far as submission type is concerned, throughout our analysis, we have worked under the assumption, confirmed by SRUC VS staff, that PMFA submissions should require transportation to a DSC by the animal keeper, whereas non-PMFA submissions typically arrive by post. This increases the likelihood of a relationship between distance and submission rate for PMFA submissions. The differences in density of submissions and proportion of holdings submitting show that in general, as distance to the closest DSC increases, a submission becomes less likely. A similar relationship is found with all submissions, but the decrease in proportion submitting is less severe. This relationship of distance, in conjunction with holding density may help to explain the lower submission rates at the two most northerly centres, Inverness and Thurso. For example, Inverness DSC has fewer than 25% of holdings from its catchment area within 50 km of the DSC location. We used 50 km as our assessment distance of the denominator for the overall network and individual DSCs as it had been stipulated for the initial evaluation. It approximates to an hour and a half complete journey time, based on an average driving speed of 40 mph, total journey distance equal to 60 miles; radius would be 30 miles i.e., approximately 50 km. The influence of distance has been noted previously. Kinnaird^17^ reported that “farmers or crofters who reported using a diagnostic laboratory were based an average of 40 miles away from the nearest SAC laboratory (DSC), compared with those who never used diagnostic laboratories, who were on average around 70 miles away.”

The importance of this relationship of distance and likelihood to submit can play an important role in the policy decisions around where to locate DSCs and how to operate the network. Previous studies have highlighted the role that farmers and veterinarians can play in disease surveillance (10) and how human connections between those involved in surveillance can be critical in identifying both a new epidemic and monitoring endemic disease (28). Whilst new technologies can help with increasing testing at an individual holding (11) PMs will continue to be required and if these need to be transported, location of the PM facilities needs consideration (19, 20). Alternative initiatives can be, and have been, implemented elsewhere to reduce the effects of distance. Carcase collection services and the establishment of “a tiered surveillance network that provides 95% of holdings and animals with access to a post-mortem facility or collection point within an hour's travel time (up from the current 50%)” were advised by the Surveillance Advisory Group (2012) for the review of services in England and Wales.

There will be other factors that influence the ultimate decision to submit, as is evidenced by those areas in the density ratio plots where more than 50% of holdings present are submitting, but the areas are not located in close proximity to a DSC. Throughout this work each holding identified was treated as identical, regardless of size. Therefore, smallholdings, crofts and commercial farms are considered the same. This decision was made because the primary aim of this evaluation was not to determine drivers for submission, but to determine whether the data quality had improved, in terms of the ability to geo-locate and identify Scottish livestock holdings that had submitted to the surveillance network.

It is quite possible that the numbers of livestock on a holding play a part in the decision to submit, as flock and herd size are so often risk factors for disease occurrence and it was stated in the 2011 Review of Veterinary Surveillance (see text footnote 7) that “There was also a significant link between the size of herds or flocks and frequency of use, with larger units making greater use of laboratories.”. There may also be other influences; previous studies have shown that smallholders and commercial farms move animals differently (29) and may need to be considered differently when it comes to biosecurity and surveillance (30). The implications for these different types of holding has already been envisioned for future scenarios (31) and may well need to be considered when exploring the reasons behind DSC usage. The interpersonal relationships with both their local veterinary practice and the local DSC are likely to be different for a smallholder, or crofter, compared to a commercial farm. Likewise, the financial incentives for disease investigations involving the DSC network, and this may be reflected in the likelihood to submit. Most of the areas where <15% of holdings have made at least one diagnostic submission of any type to the DSC network in 2013–2018 are areas that are traditionally associated with the Scottish croft system of livestock ownership and management, or are remote mountain and moorland areas.

For this re-evaluation we have used the term “coverage” to refer to the Scottish livestock holdings that have the potential to submit to the DSC network. If they were to experience a disease event, which led to a decision that further diagnostic support was warranted. This differs from the more usual use of this term as a surveillance attribute for the coverage achieved, which we term “usage”, i.e., the holdings that did submit. Ely et al. (32) explored different measures of assessment for pig submissions in four areas of England and discussed why the values obtained varied. We have assumed that the decision to submit is made at the holding-level, while recognising that there will be multiple factors that can play a part in arriving at this decision.

### Future work

For this analysis we opted to use straight-line distance to define the DSC catchment areas and the distance to the closest DSC. This does lead to some potential anomalies, most notably when this leads to livestock holdings from the same island being allocated to two different DSCs. It is more likely that the geographical and transport routes will be similar for the whole island. Remote and rural transport routes are often defined by the topography; this may be a contributing factor to the number of holdings that never submit to their closest DSC, when that closest DSC is established by straight-line distance. In addition to these topographical and transport influences, there may also be individual farmer or veterinarian preference and that of submission type. These geographical differences are likely to be one cause of the observed bimodal distributions (Figures 6 and Supplementary Figure SM12). An element of future work could include conducting a thorough route analysis (26, 27), as these techniques may enable a better understanding of any transport related differences in submission rates.

Now that we have confidence in the submissions data set, future work could also include a thorough investigation into the drivers for submission. However, there remains the difficulty of how to assign an accurate value to number of animals on each holding at each point in time and as the analysis was conducted over a five-year period, it also raises the question of how to summarise herd size over time.

## Conclusions

Diagnostic services serving agricultural communities are a mainstay of many surveillance systems. However, there are questions about how these networks should be set up or existing networks modified, how representative they are of the whole population at risk and whether they indeed need to be. Outputs from such passive surveillance systems can be hard to interpret and extrapolate as it is often suspected that only a proportion of those eligible to submit do so and any potential for bias in the system is poorly described. This highlights the need for regular evaluation. With our evaluation of the DSC network, we have established a baseline reference footprint that is of use to both the surveillance provider and science-policy advisors, who fund the network for surveillance purposes. This baseline can be used in future assessments of the network. These could examine how changes in the network that were implemented from 2019 onwards, and other shocks such as the UK's exit from the European Union and pandemic restrictions, have affected usage of the DSC network. We also now have sufficient confidence in the data to investigate possible drivers for submission, if that knowledge is required. In 2022/23, a veterinary surveillance intelligence unit is being established to make improved use of additional data sources, strengthen links with users of the network and ensure that it acts as a surveillance multiplier with an overall picture of livestock disease and trends in Scotland. In addition a new LIMS is being introduced. Ideally, with these two new initiatives commencing, we would now begin to re-evaluate the footprint from the next 5 year period (2019 to 2023), as a prelude to subsequently evaluating the impact of these further changes.

## Data availability statement

The data analysed in this study is subject to the following licenses/restrictions: The data generated and analysed by this study are available from APHA, ScotEID, and SRUC VS but restrictions apply to the availability of these data, which were used under license for the current study, and so are not publicly available. Data are however available from the authors upon reasonable request and with permission of APHA, ScotEID, and SRUC VS. Requests to access these datasets should be directed to ST, sue.tongue@sruc.ac.uk.

## Author contributions

AD carried out the data preparation, conducted most of the analysis, and interpreted the data. JE contributed to the analysis and interpretation of the data. FB extracted the submission data and contributed to the interpretation of the data. AJ and JS extracted the holding dataset. ST conceived the idea, sourced the funding, led, managed the project including setting the direction, and contributed to the interpretation of the data. All authors contributed to revision and drafts of the manuscript and approved the final version.

## Footnotes

^1^ https://assets.publishing.service.gov.uk/government/uploads/system/uploads/attachment_data/file/869173/uksf-animal-health-surveillance.pdf

^2^ https://www.sruc.ac.uk/all-news/implementing-a-new-model-of-disease-surveillance/

^3^ http://apha.defra.gov.uk/vet-gateway/surveillance/scanning/vida.htm

^4^ http://apha.defra.gov.uk/vet-gateway/surveillance/diagnostic/national-network.htm

^5^ https://www.gov.ie/en/service/7f9f7-laboratory-services/#regional-veterinary-laboratories

^6^ https://www.iaea.org/services/networks/vetlab

^7^ https://webarchive.nrscotland.gov.uk/3000/https://www.gov.scot/Resource/Doc/362344/0122619.pdf

^8^Tongue et al. (2015), *The SAC Consulting Veterinary Services Disease Surveillance Centre network - what parts does it reach?*, unpublished internal peer reviewed report for Scottish Government, available on request.

^9^ https://www.gov.uk/guidance/register-land-you-use-to-keep-livestock#holding-meaning-and-the-area-it-can-cover

^10^ https://www.gov.scot/collections/june-scottish-agricultural-census/

^11^ https://secure.services.defra.gov.uk/wps/portal/ctso

^12^ https://www.gov.uk/government/organisations/animal-and-plant-health-agency

^13^ https://www.scoteid.com/

^14^ http://apha.defra.gov.uk/documents/surveillance/diseases/lddg-pop-report-sheep2021.pdf

^15^ https://www.gov.scot/publications/results-scottish-agricultural-census-june-2021/pages/3/

^16^ https://www.gov.scot/publications/scottish-bvd-eradication-scheme/

## References

[1] Shahab SS. Finding Value in the Evaluation of Public Health Syndromic Surveillance Systems From a Policy Perspective in Finding Value in the Evaluation of Public Health Syndromic Surveillance Systems From a Policy Perspective. Alberta: Alberta Health Services (2009).

[2] Peyre M Hoinville L Njoroge J Cameron A Traon D Goutard F. The RISKSUR EVA tool (Survtool): A tool for the integrated evaluation of animal health surveillance systems. Prev Vet Med. (2019) 173:104777. 10.1016/j.prevetmed.2019.104777 31731037

[3] Jann W Wegrich K. Theories of the Policy Cycle In:F. Fischer and G Miller, editors, *Handbook of Public Policy Analysis*, New York, NY: Routledge. (2007).

[4] Calba C Goutard FL Hoinville L Hendrikx P Lindberg A Saegerman C. Surveillance systems evaluation: a systematic review of the existing approaches. BMC Public Health. (2015) 15:448. 10.1186/s12889-015-1791-5 25928645 PMC4418053

[5] Hendrikx P Gay E Chazel M Moutou F Danan C Richomme C. an assessment tool of epidemiological surveillance systems in animal health and food safety. Epidemiol Infect. (2011) 139:1486–96. 10.1017/S0950268811000161 21385516

[6] Drewe JA Hoinville LJ Cook AJ Floyd T Gunn G Stärk KD. a new framework for the evaluation of animal health surveillance. Transbound Emerg Dis. (2015) 62:33–45. 10.1111/tbed.12063 23414450

[7] Dufour B. Technical and economic evaluation method for use in improving infectious animal disease surveillance networks. Vet Res. (1999) 30:27–37. 10081110

[8] Klaucke DN. Evaluating Public Health Surveillance Systems. In W. Halperin, E. Baker and R. Monson, editors *Public Health Surveillance*, New York, NY: Van Nostrand Reinhold (1992), p. 26–41.25032347

[9] Hoinville LJ Alban L Drewe JA Gibbens JC Gustafson L Häsler B. Proposed terms and concepts for describing and evaluating animal-health surveillance systems. Prev Vet Med. (2013) 112:1–12. 10.1016/j.prevetmed.2013.06.006 23906392

[10] Gates MC Earl L Enticott G. Factors influencing the performance of voluntary farmer disease reporting in passive surveillance systems: a scoping review. Prev Vet Med. (2021) 196:105487. 10.1016/j.prevetmed.2021.105487 34507237

[11] Gates MC Holmstrom LK Biggers KE Beckham TR. Integrating novel data streams to support biosurveillance in commercial livestock production systems in developed countries: challenges and opportunities. Front Pub Health. (2015) 3:74. 10.3389/fpubh.2015.00074 25973416 PMC4411973

[12] Lawson B Petrovan SO Cunningham AA. Citizen science and wildlife disease surveillance. Ecohealth. (2015) 12:693–702. 10.1007/s10393-015-1054-z 26318592

[13] Toolan DP Mitchell G Searle K Sheehan M Skuce PJ Zadoks RN. Bovine and ovine rumen fluke in Ireland - Prevalence, risk factors and species identity based on passive veterinary surveillance and abattoir findings. Vet Parasitol. (2015) 212:168–74. 10.1016/j.vetpar.2015.07.040 26298508

[14] Vanclay F Enticott G. The role and functioning of cultural scripts in farming and agriculture. Sociol Ruralis. (2011) 51:256–71. 10.1111/j.1467-9523.2011.00537.x

[15] Brugere C Onuigbo DM Morgan KL. People matter in animal disease surveillance: challenges and opportunities for the aquaculture sector. Aquaculture. (2017) 467:158–69. 10.1016/j.aquaculture.2016.04.012

[16] Enticott G Earl L Gates MC A. systematic review of social research data collection methods used to investigate voluntary animal disease reporting behaviour. Transbound Emerg Dis. (2021) 69:2573–87. 10.1111/tbed.14407 34843177

[17] Sawford K Vollman AR Stephen C. A focused ethnographic study of alberta cattle veterinarians' decision making about diagnostic laboratory submissions and perceptions of surveillance programs. PLoS ONE. (2013) 8:e64811. 10.1371/journal.pone.0064811 23741397 PMC3669388

[18] Robinson PA Epperson WB Huston CL Pace LW Wills RW Cosby AG. Factors influencing diagnostic sample submission by food animal veterinarians in Mississippi. Vet Ital. (2012) 48:31–9. Available online at: https://www.izs.it/vet_italiana/2012/48_1/31.htm 22485000

[19] Zühlke I Berezowski J Bodmer M Küker S Göhring A Rinaldi F. Factors associated with cattle necropsy submissions in Switzerland, and their importance for surveillance. Prev Vet Med. (2021) 187:105235. 10.1016/j.prevetmed.2020.105235 33453476

[20] McFarland L Macken-Walsh Á Claydon G Casey M Douglass A McGrath G. Irish dairy farmers' engagement with animal health surveillance services: Factors influencing sample submission. J Dairy Sci. (2020) 103:10614–27. 10.3168/jds.2019-17889 32861485

[21] Duncan AJ Reeves A Gunn GJ Humphry RW. Quantifying changes in the British cattle movement network. Prev Vet Med. (2022) 198:105524. 10.1016/j.prevetmed.2021.105524 34775127

[22] QGIS.org. QGIS Geographic Information System. QGIS Association (2022). Available online at: https://qgis.org/en/site/getinvolved/faq/index.html#how-to-cite-qgis

[23] R Core Team. R: A Language and Environment for Statistical Computing. Vienna (2022).

[24] Wickham H Averick M Bryan J Chang W McGowan LD François R. Welcome to the Tidyverse. J Open Source Software. (2019) 4:1686. 10.21105/joss.01686

[25] Villanueva RA Chen ZJ. ggplot2: Elegant Graphics for Data Analysis. New York, NY: Springer-Verlag (2016).

[26] Morgan M Lovelace R. Ravel flow aggregation: Nationally scalable methods for interactive and online visualisation of transport behaviour at the road network level. Environ Plan Urban Anal City Sci. (2021) 48:1684–96. 10.1177/2399808320942779

[27] Lovelace R Félix R Carlino D. Jittering: A computationally efficient method for generating realistic route networks from origin-destination data. Findings. (2022) 8:33873. 10.32866/001c.33873

[28] Gilbert WH Häsler BN Rushton J. Influences of farmer and veterinarian behaviour on emerging disease surveillance in England and Wales. Epidemiol Infect. (2014) 142:172–86. 10.1017/S0950268813000484 23527498 PMC9152612

[29] Porphyre T Boden LA Correia-Gomes C Auty HK Gunn GJ Woolhouse ME. How commercial and non-commercial swine producers move pigs in Scotland: a detailed descriptive analysis. BMC Vet Res. (2014) 10:140. 10.1186/1746-6148-10-140 24965915 PMC4082416

[30] Hernández-Jover M Hayes L Woodgate R Rast L Toribio JAL. Animal health management practices among smallholder livestock producers in Australia and their contribution to the surveillance system. Front Vet Sci. (2019) 6:191. 10.3389/fvets.2019.00191 31275950 PMC6591531

[31] Boden LA Auty H Reeves A Rydevik G Bessell P McKendrick IJ. Animal health surveillance in Scotland in 2030: using scenario planning to develop strategies in the context of brexit. Front Vet Sci. (2017) 4:201. 10.3389/fvets.2017.00201 29230402 PMC5711829

[32] Ely ER Nicholson RE Snow LC Strugnell BW Williamson SM Milnes AS. Evaluation of methods for measuring coverage and representativeness of an early-warning disease surveillance system. Vet Record. (2012) 171:423–423. 10.1136/vr.100854 23015726

