# Corrigendum: Evaluations of the disease surveillance centre network in Scotland: what parts has it reached?

**DOI:** 10.3389/fvets.2023.1192445

**Published:** 2023-04-12

**Authors:** Andrew J. Duncan, Jude I. Eze, Franz Brülisauer, Julie M. Stirling, Amy Jennings, Sue C. Tongue

**Affiliations:** ^1^Centre for Epidemiology and Planetary Health, Department of Veterinary and Animal Science, Northern Faculty, Scotland's Rural College (SRUC), Inverness, United Kingdom; ^2^UHI Inverness, University of the Highlands and Islands, Inverness, United Kingdom; ^3^Biomathematics and Statistics Scotland, Edinburgh, United Kingdom; ^4^SRUC Veterinary Services, Inverness, United Kingdom; ^5^The Royal (Dick) School of Veterinary Studies and The Roslin Institute, University of Edinburgh, Edinburgh, United Kingdom

**Keywords:** disease surveillance, evaluation, network, veterinary, farmer, passive surveillance, livestock

In the published article, there was an error. Some typographical errors were in the abstract that were not picked up during the proofing stage.

A correction has been made to **Abstract**. This section previously stated:

“Regular evaluation is a prerequisite for systems that provide surveillance of animal populations. Scotland's Rural College Veterinary vices' Disease Surveillance Centre (DSC) network plays an integral part in surveillance to detect new and re-emerging threats within animal populations, predominantly livestock. In ronse to surveillance reviews and proposed changes to the network, an initial evaluation of diagnostic submissions data in 2010 to mid-2012 established a baseline “footprint,” while highlighting challenges with the data. In this recenaluation for the period 2013–2018, we developed a new denominator using a combination of agricultural census and movement data, to identify relevant holdings more accurately. Iterative discussions between those processing submissions data ahose involved in collection at source took place to understand the intricacies of the data, establish the most appropriate dataset, and develop the processes required to optimize the data extraction and cleansing. The subsequent descriptive analysis identifies the number of diatic submissions, the number of unique holdings making submissions to the network and shows that both the surrounding geographic region of, and maximum dise to the closest DSC vary greatly between centers. Analysis of those submissions classed as farm animal post-mortems also highlights the effect of distance to the closest DSC. Whether specific differences between the time periods are due to changes in the behavior of the submitting holdior the data extraction and cleaning processes was difficult to disentangle. However, with the improved techniques producing better data to work with, a new baseline foot prior the network has been created. This provides information that can help policy makers and surveillance providers make decisions about service provision and evaluate the impact of future changes. Additionally, thtputs of these analyses can provide feedback to those employed in the service, providing evidence of what they are achieving and why changes to data collection processes and ways of working are being made. In a different setting, er data will be available and different challenges may arise. However, the fundamental principles highlighted in these evaluations and the solutions developed should be of interest to any surveillance providers generating similar diagnostic data”.

The corrected section appears below:

“Regular evaluation is a prerequisite for systems that provide surveillance of animal populations. Scotland's Rural College Veterinary Services' Disease Surveillance Centre (DSC) network plays an integral part in surveillance to detect new and re-emerging threats within animal populations, predominantly livestock. In response to surveillance reviews and proposed changes to the network, an initial evaluation of diagnostic submissions data in 2010 to mid-2012 established a baseline “footprint,” while highlighting challenges with the data. In this recent evaluation for the period 2013–2018, we developed a new denominator using a combination of agricultural census and movement data, to identify relevant holdings more accurately. Iterative discussions between those processing submissions data and those involved in collection at source took place to understand the intricacies of the data, establish the most appropriate dataset, and develop the processes required to optimise the data extraction and cleansing. The subsequent descriptive analysis identifies the number of diagnostic submissions, the number of unique holdings making submissions to the network and shows that both the surrounding geographic region of, and maximum distance to the closest DSC vary greatly between centres. Analysis of those submissions classed as farm animal post-mortems also highlights the effect of distance to the closest DSC. Whether specific differences between the time periods are due to changes in the behavior of the submitting holdings or the data extraction and cleaning processes was difficult to disentangle. However, with the improved techniques producing better data to work with, a new baseline footprint for the network has been created. This provides information that can help policy makers and surveillance providers make decisions about service provision and evaluate the impact of future changes. Additionally, the outputs of these analyses can provide feedback to those employed in the service, providing evidence of what they are achieving and why changes to data collection processes and ways of working are being made. In a different setting, other data will be available and different challenges may arise. However, the fundamental principles highlighted in these evaluations and the solutions developed should be of interest to any surveillance providers generating similar diagnostic data.”

The authors apologize for this error and state that this does not change the scientific conclusions of the article in any way. The original article has been updated.

