# Supplementary material for: Evaluations of the Disease Surveillance Centre network in Scotland: What parts has it reached?
Source: Front Vet Sci. 2023 Feb 21;10:1099057. doi: 10.3389/fvets.2023.1099057 (PMC9988905; doi:10.3389/fvets.2023.1099057)
Supplement: Supplementary file 1 [file Data_Sheet_1.pdf]

Evaluations of the Disease Surveillance Centre Network in Scotland: What parts has it reached?

Supplementary Material

1 Holdings

Figures SM1 – SM3 below show the spatial distribution of all Scottish holdings recorded as having either cattle (SM1), sheep (SM2) or pigs (SM3) on them during 2013 – 2018.

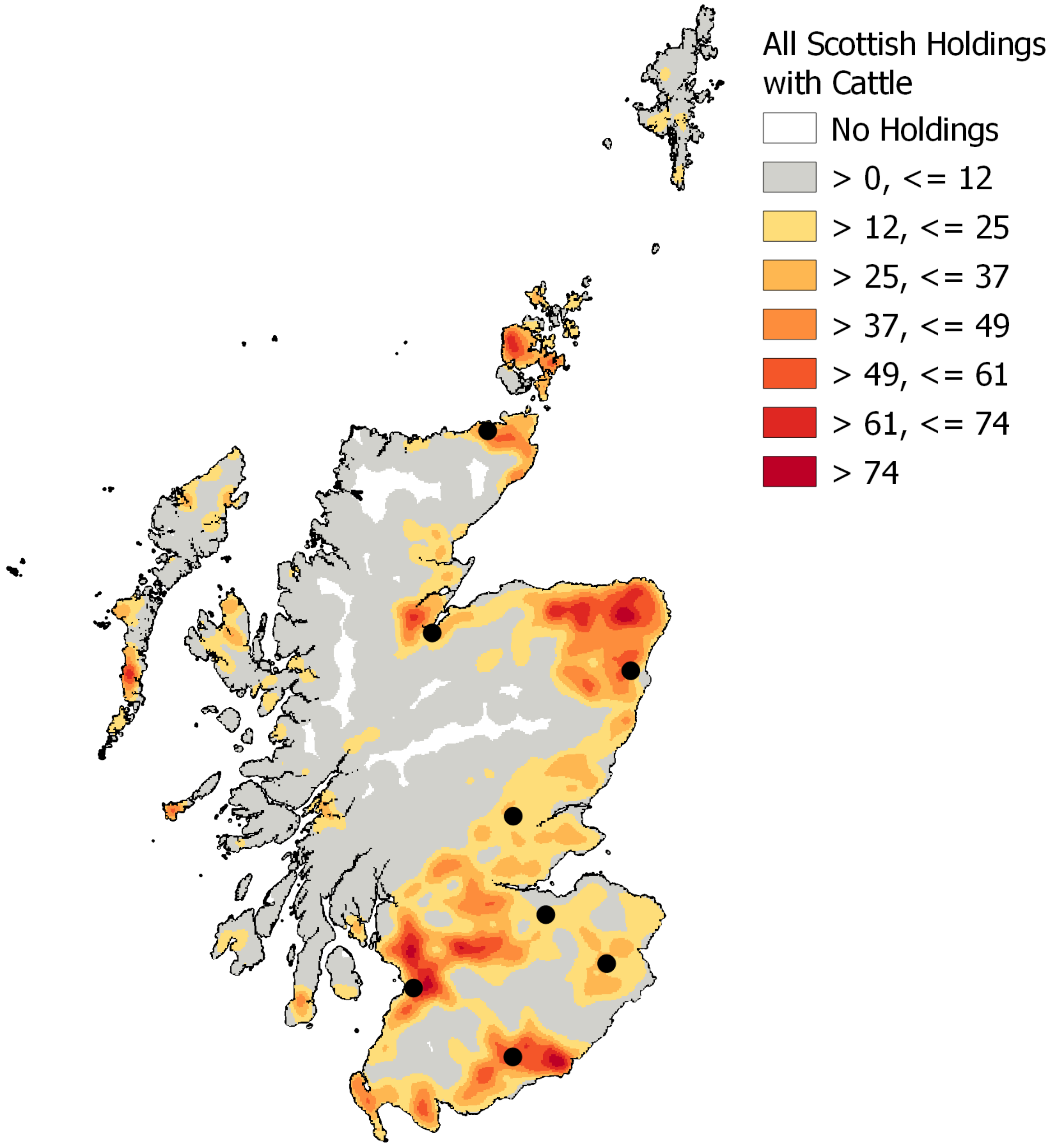

Figure SM1: All Scottish livestock holdings with cattle 2013-2018 inclusive, as defined from census and movement data - Kernel density 10 km radius, number of holdings per 10km-square - with the locations of the eight DSCs (black spots)

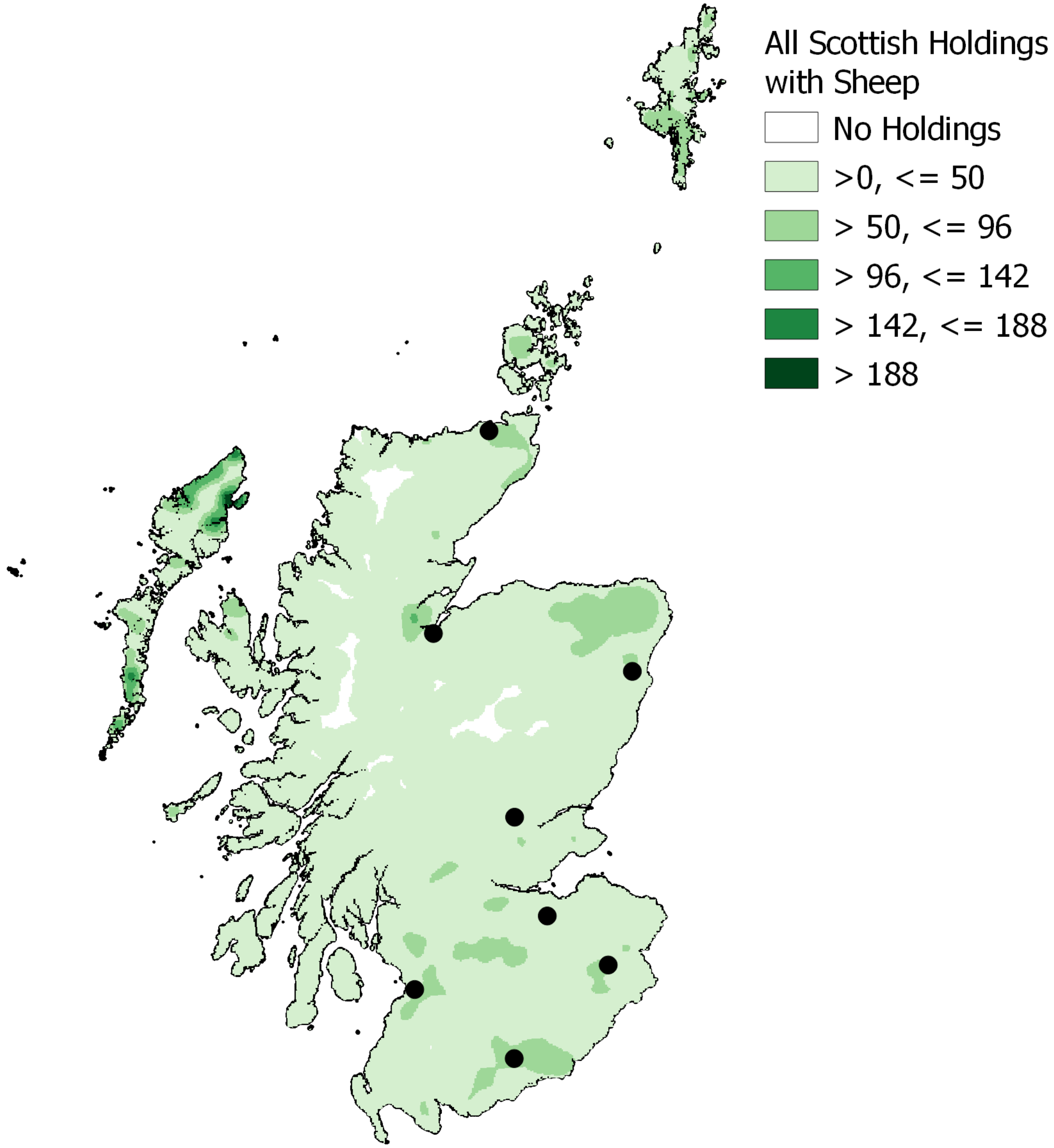

Figure SM2: All Scottish livestock holdings with sheep 2013-2018 inclusive, as defined from census and movement data - Kernel density 10 km radius, number of holdings per 10km-square - with the locations of the eight DSCs (black spots)

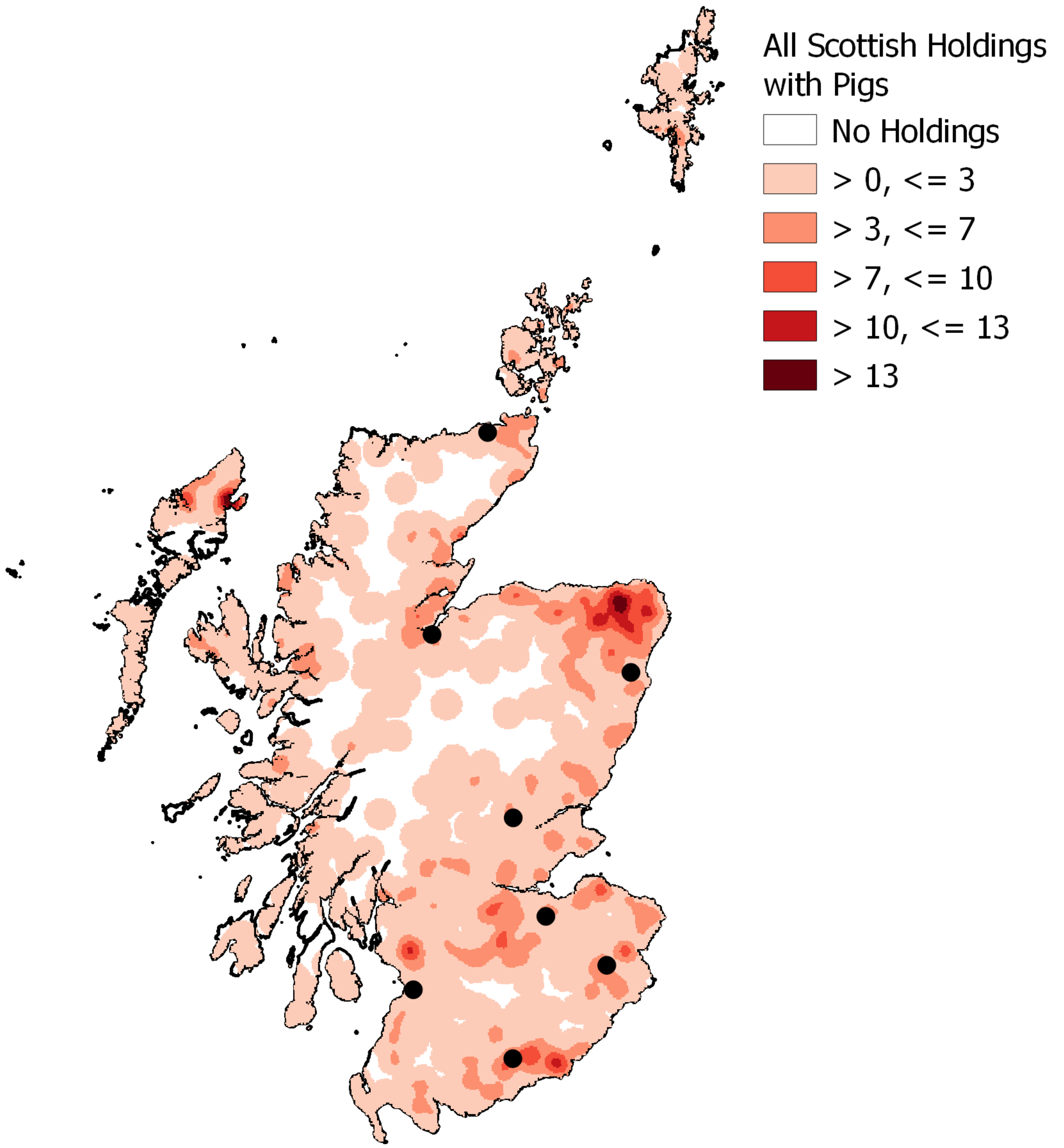

Figure SM3: All Scottish livestock holdings with pigs 2013-2018 inclusive, as defined from census and movement data - Kernel density 10 km radius, number of holdings per 10km-square - with the locations of the eight DSCs (black spots).

Figures SM4 – SM11 show the cumulative frequency of the distance from each Scottish livestock holding in the catchment area of each Disease Surveillance Centre (DSC), to that respective DSC . The distribution for each catchment area is quite different with the most northerly centres, Inverness and Thurso, having the greatest maximum distance from a holding to a disease centre.

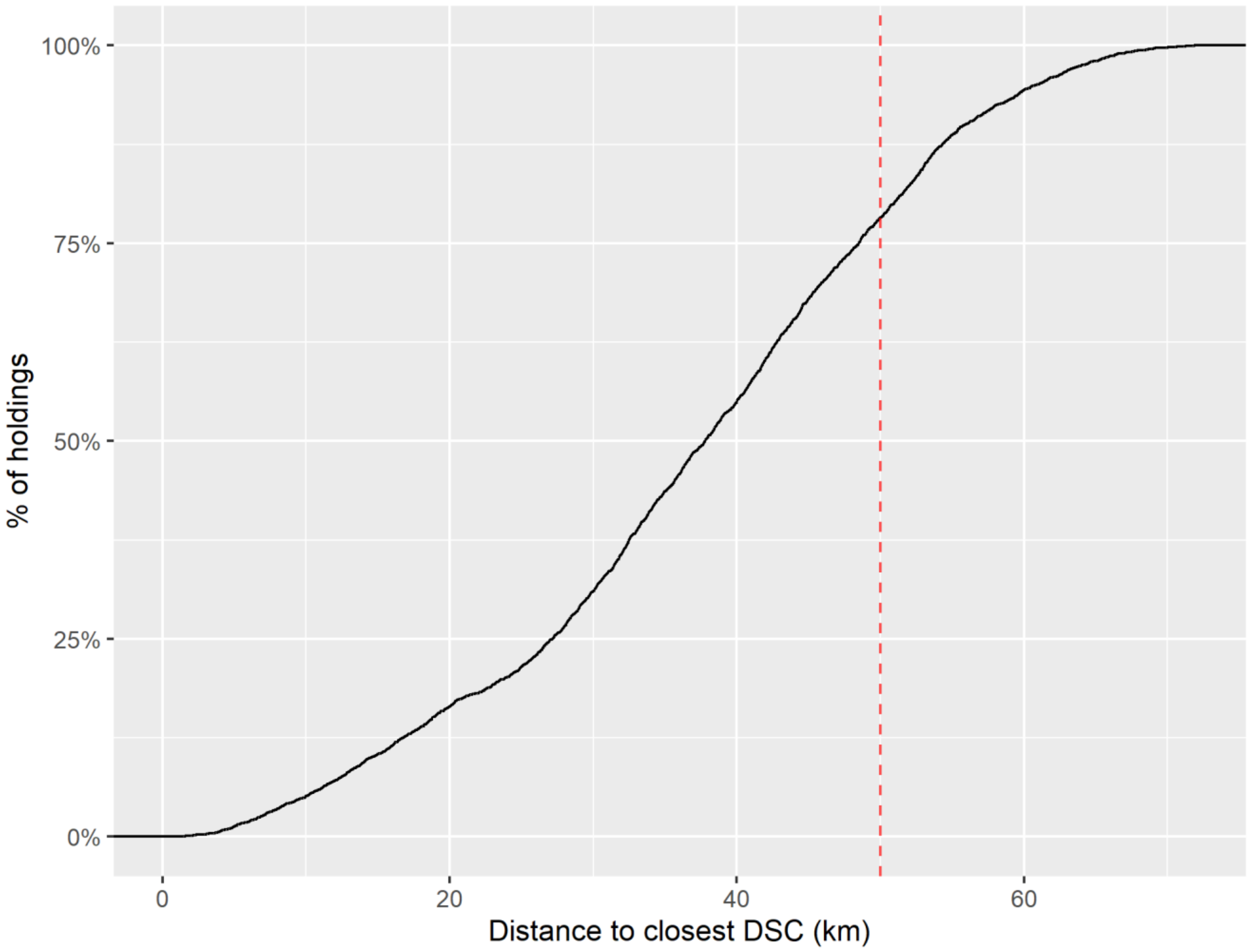

Figure SM4: Cumulative Frequency of Distance from Scottish livestock holdings within the catchment of Aberdeen DSC, to the DSC itself.

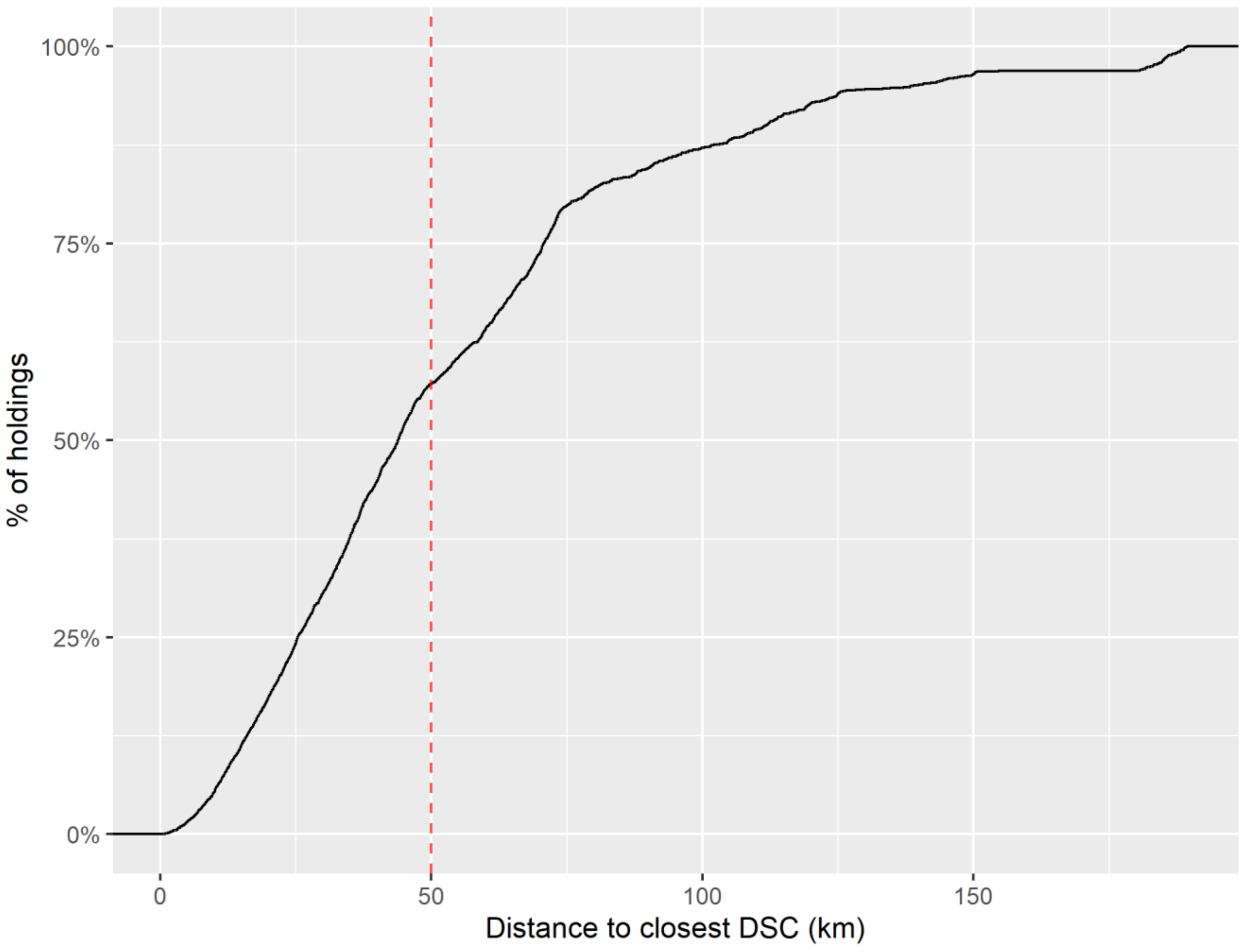

Figure SM5: Cumulative Frequency of Distance from Scottish livestock holdings within the catchment of Ayr DSC, to the DSC itself.

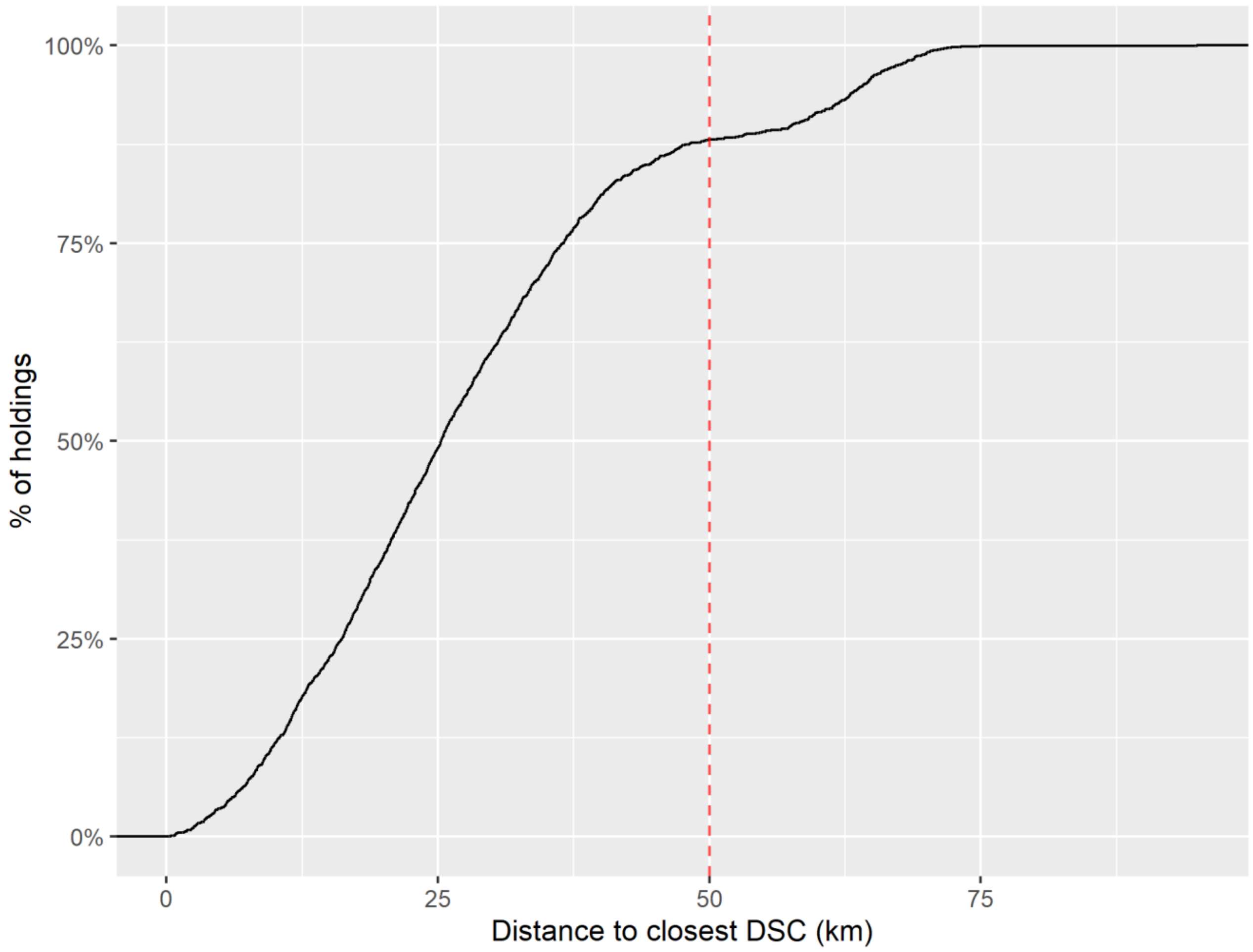

Figure SM6: Cumulative Frequency of Distance from Scottish livestock holdings within the catchment of Dumfries DSC, to the DSC itself.

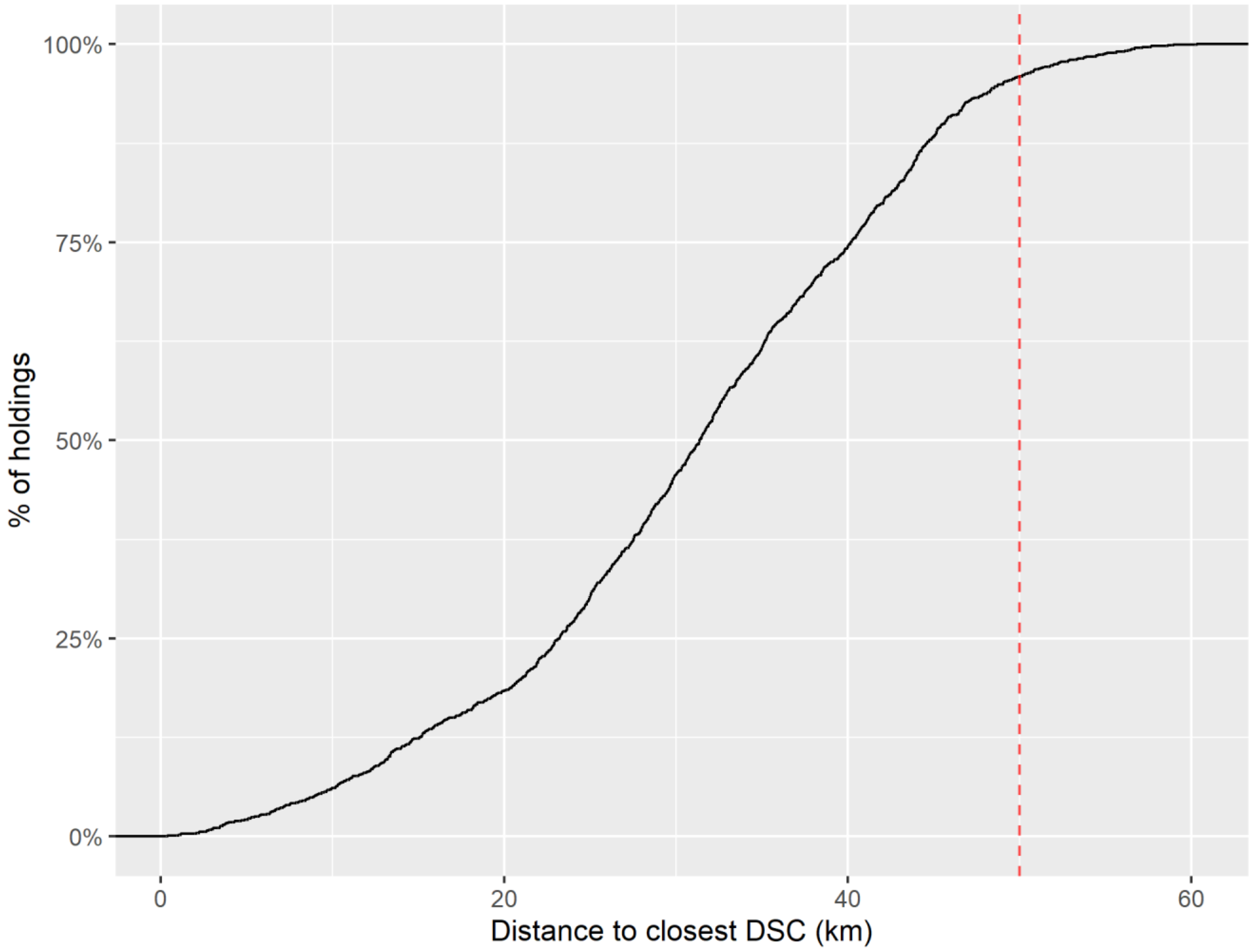

Figure SM7: Cumulative Frequency of Distance from Scottish livestock holdings within the catchment of Edinburgh DSC, to the DSC itself.

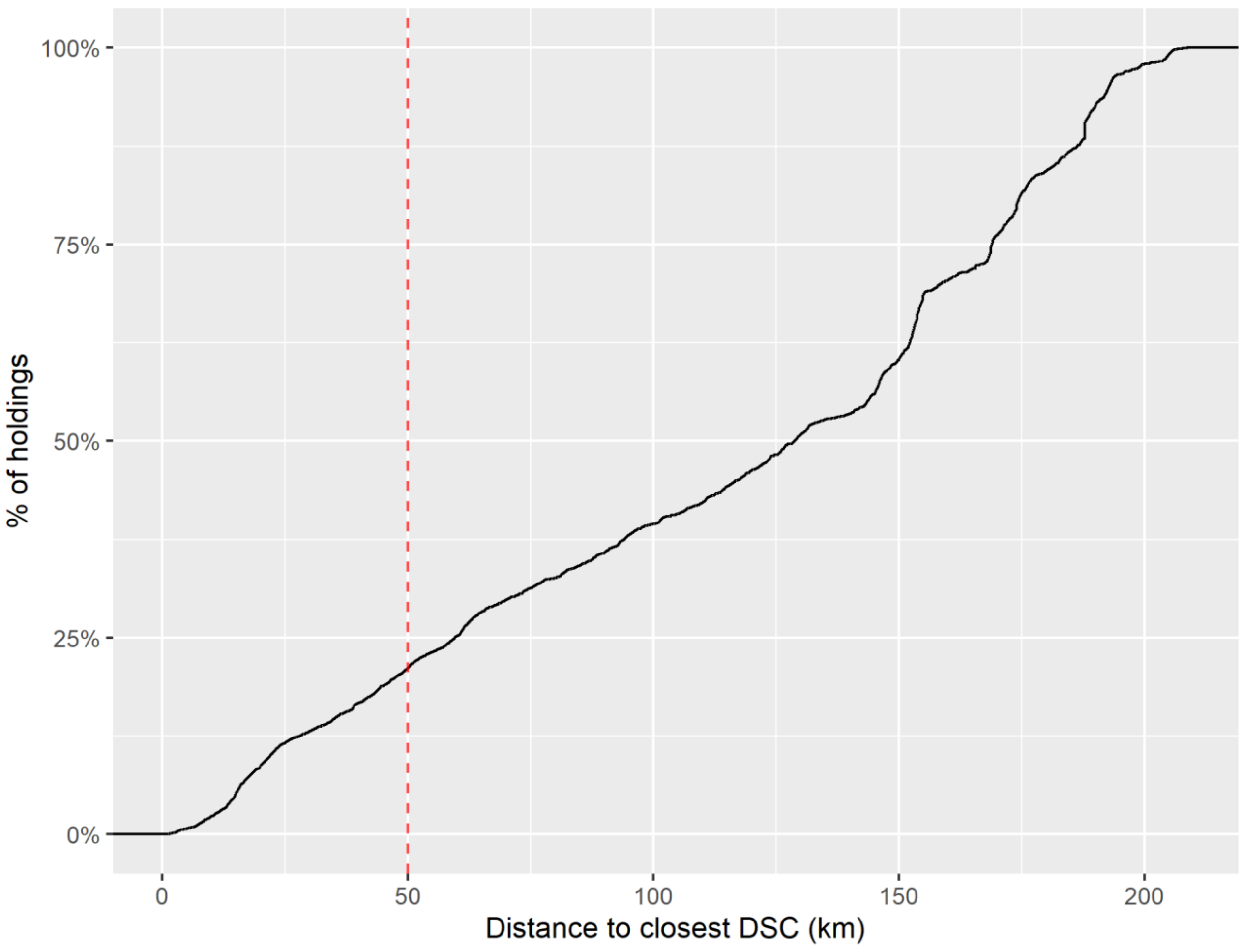

Figure SM8: Cumulative Frequency of Distance from Scottish livestock holdings within the catchment of Inverness DSC, to the DSC itself.

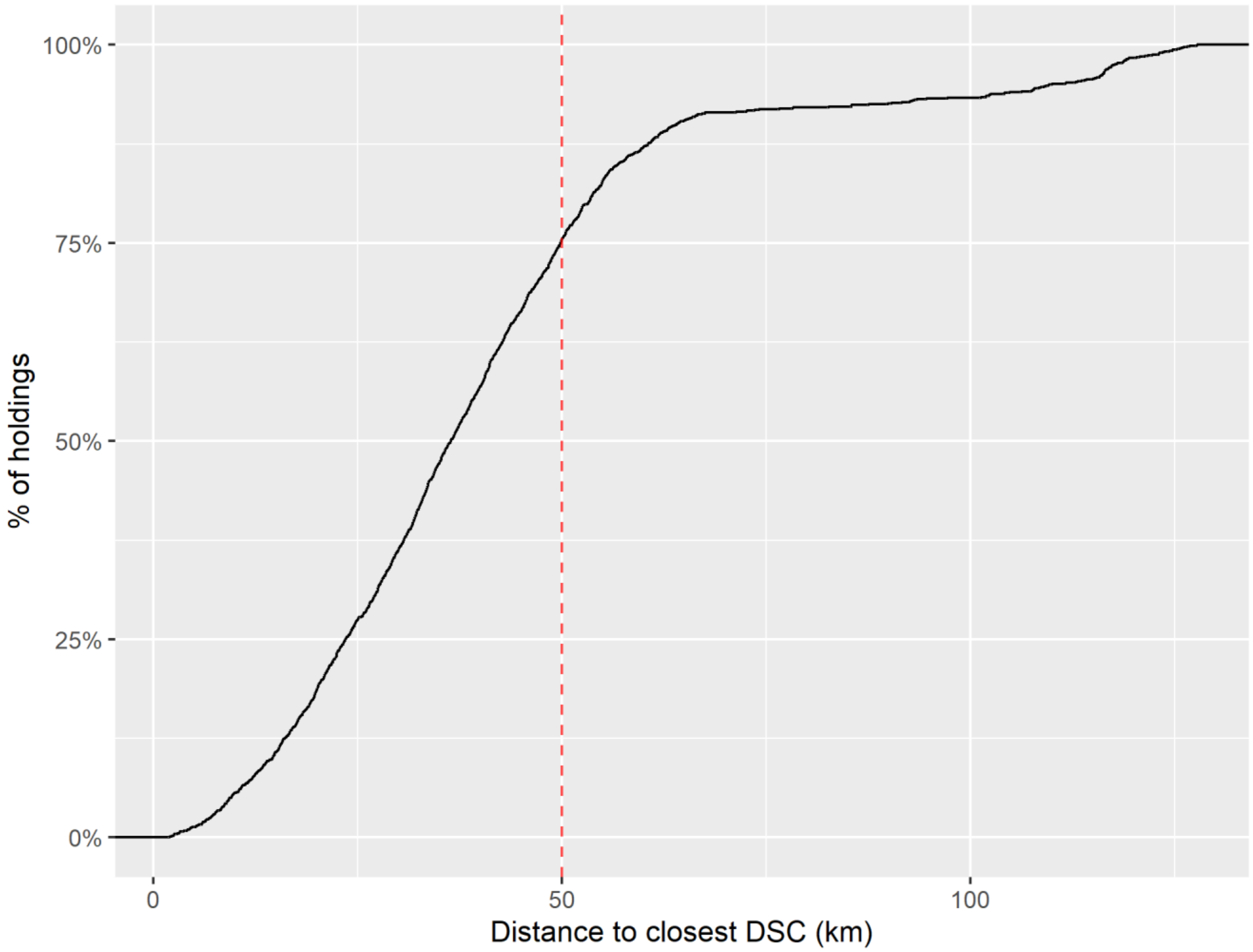

Figure SM9: Cumulative Frequency of Distance from Scottish livestock holdings within the catchment of Perth DSC, to the DSC itself.

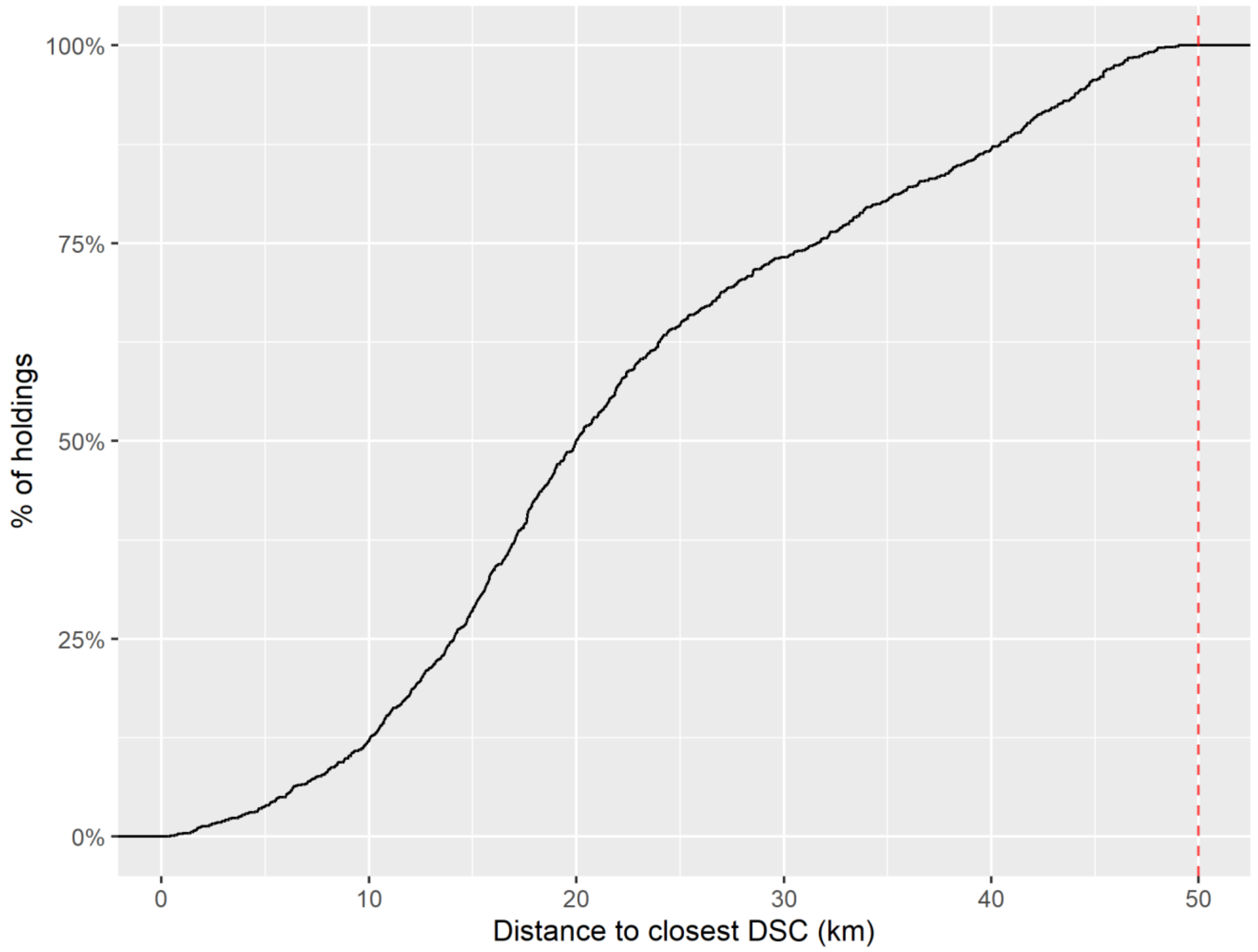

Figure SM10: Cumulative Frequency of Distance from Scottish livestock holdings within the catchment of St. Boswells DSC, to the DSC itself.

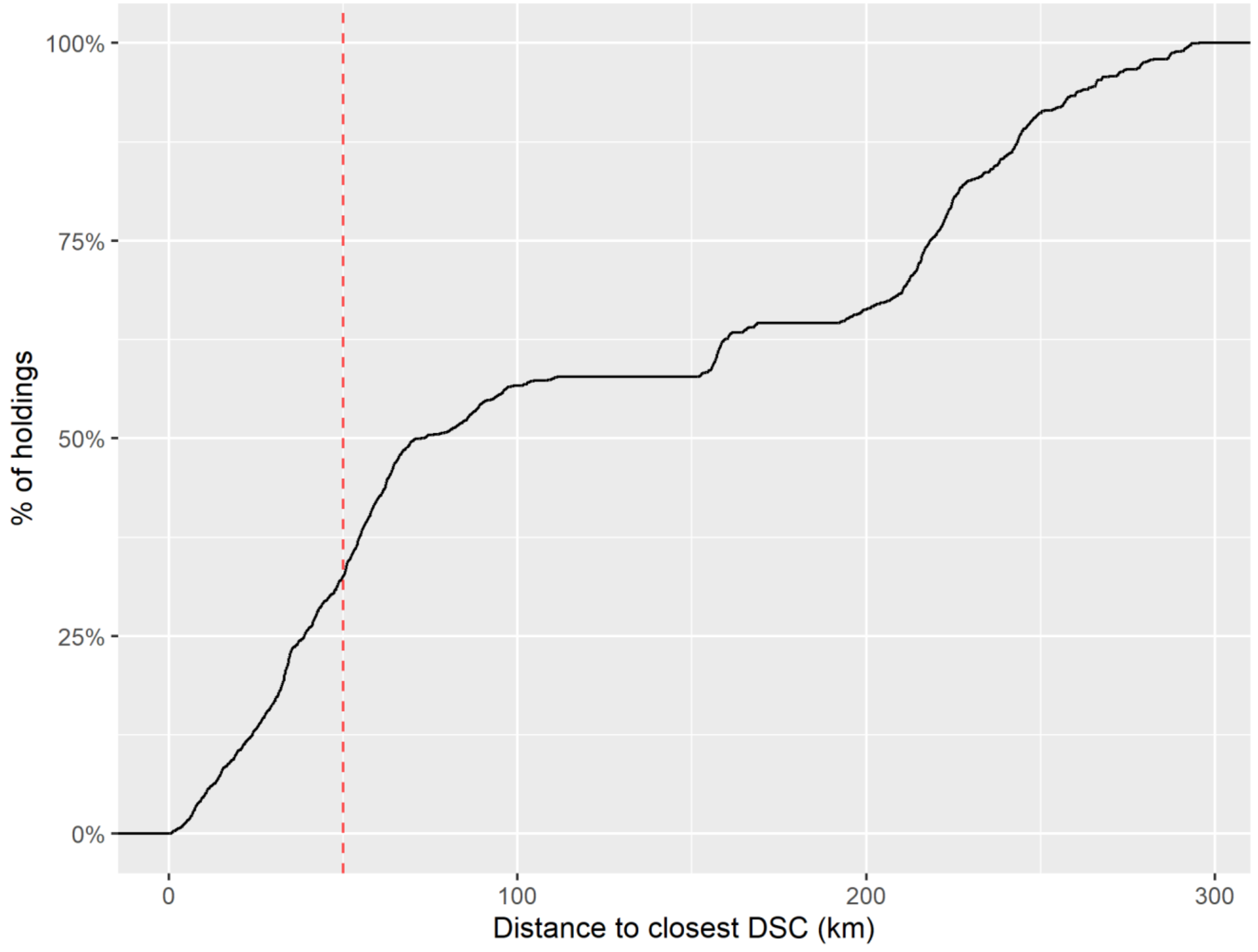

Figure SM11: Cumulative Frequency of Distance from Scottish livestock holdings within the catchment of Thurso DSC, to the DSC itself.

2 Submission records – the numerator

Tables SM1 and SM2 show the proportion of livestock holdings, either all Scottish or within the catchment of a particular DSC that made either a diagnostic or a diagnostic post-mortem farm animal (PMFA) submission to the DSC network respectively.

Table SM1: Distributions of Scottish livestock holdings by named catchment area - aspects relating to diagnostic submissions 2013 - 2018 (2010 to mid-2012). Final data sets: 40,564 (34, 035) diagnostic submission records.

	% holdings within named catchment area who made at least one submission to the network	Number of holdings, who made at least one submission to the network, that are located in named catchment area	% of all livestock holdings, who made at least one submission to the network, who are located in named catchment area	% of holdings that submitted, which – in the named catchment area – submitted at least once to their closest centre
**All Scottish CPHs**	**26.3 (23.4)**	**6,322 (5,095)**	**26.3 (23.4)**	**90.8 (89.9)**
Aberdeen	29.6 (28.2)	1014 (904)	4.21 (4.1)	98.0 (97.6)
Ayr	35.5 (32.4)	1213 (1049)	5.04 (4.8)	81.7 (85.1)
Dumfries	39.2 (40.5)	807 (746)	3.35 (3.4)	98.1 (98.3)
Edinburgh	25.1 (27.5)	418 (425)	1.74 (2.0)	79.4 (79.8)
Inverness	13.8 (9.4)	894 (525)	3.72 (2.4)	89.7 (83.6)
Perth	35.2 (33.9)	829 (717)	3.45 (3.3)	91.7 (90.5)
St Boswell’s	33.3 (29.4)	375 (284)	1.56 (1.3)	94.7 (91.5)
Thurso	22.0 (13.4)	773 (445)	3.21 (2.0)	92.8 (86.3)

Table SM2: Distributions of Scottish livestock holdings by named catchment area - aspects relating to diagnostic post-mortem of farm animal submissions 2013 - 2018 (2010 to mid-2012). Final data sets: 8,342 (5,886) diagnostic PMFA submission records

	% holdings within named catchment area who made at least one submission to the network	Number of holdings, who made at least one submission to the network, that are located in named catchment area	% of all livestock holdings who made at least one submission to the network, who are located in named catchment area	% of holdings that submitted, which – in the named catchment area – submitted at least once to their closest centre
**All Scottish CPHs**	**12.2 (9.5)**	**2,937 (2,059)**	**12.2 (9.5)**	**93.9 (94.5)**
Aberdeen	13.3 (10.8)	455 (346)	1.89 (1.6)	98.2 (98.2)
Ayr	16.2 (12.4)	553 (403)	2.30 (1.9)	89.3 (92.1)
Dumfries	21.8 (19.4)	448 (358)	1.86 (1.7)	98.2 (98.3)
Edinburgh	12.0 (10.7)	199 (166)	0.83 (0.8)	75.4 (81.3)
Inverness	4.65 (3.0)	301 (167)	1.25 (0.8)	92.7 (90.4)
Perth	19.6 (15.4)	461 (326)	1.92 (1.5)	96.5 (96.9)
St Boswell’s	23.8 (15.1)	270 (145)	1.12 (0.7)	98.9 (96.6)
Thurso	7.10 (4.4)	250 (148)	1.04 (0.7)	94.4 (94.6)

Figure SM12 shows the distribution of the proportion of all Scottish livestock holdings making submissions to the DSC network as the distance to their closest DSC increases.

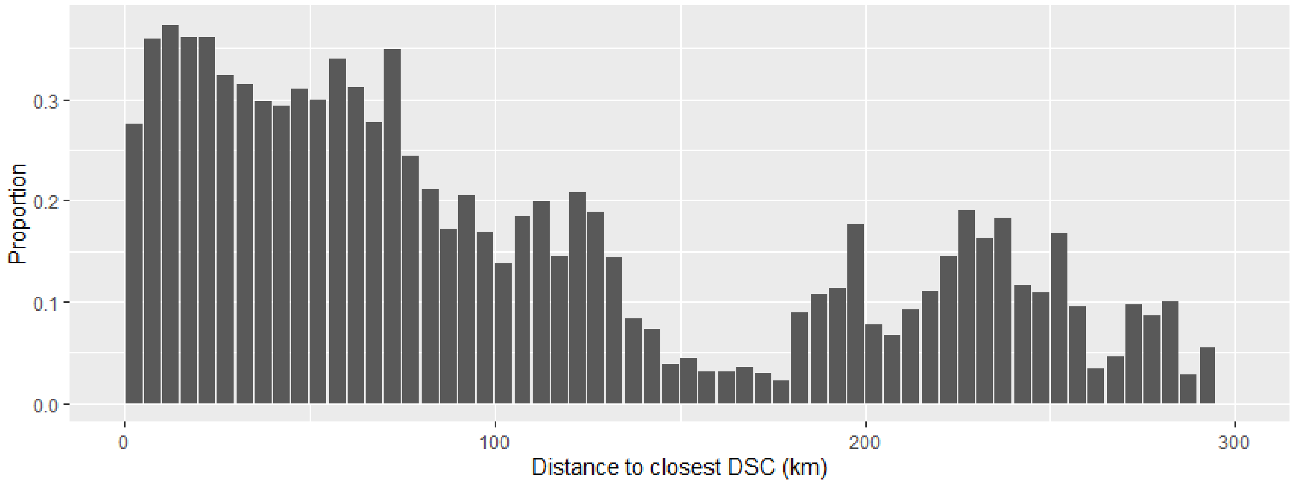

Figure SM12: Proportion of all Scottish livestock holdings making at least one submission split by the distance to their closest DSC.

Figure SM13 shows that most holdings submit only to their closest DSC but that there are small proportions who submit mostly to their closest DSC and some who never submit to their closest centre. The distribution of these across the DSC network is shown in Table SM3.

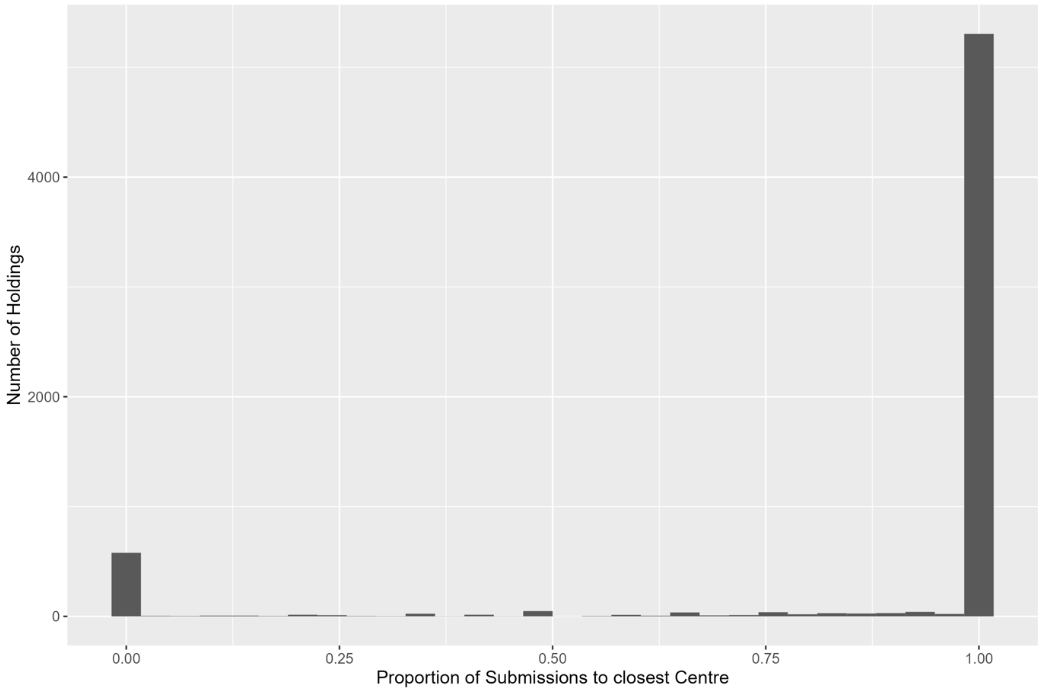

Figure SM13: The proportion of submissions per submitting Scottish livestock holding that were made to the holding's closest DSC.

Table SM3 shows the distribution of the submissions to the DSC network from the 547 Scottish livestock holdings that never submitted to the closest centre in the evaluation period, 2013 - 2018.

Table SM3: The number of submissions from the 547 holdings that did not submit at all to their closest DSC in the evaluation period (2013 - 2018), stratified by the location of their closest DSC.

Closest Centre	Centre Submitted To							
	Aberdeen	Ayr	Dumfries	Edinburgh	Inverness	Perth	St. Boswells	Thurso
Aberdeen	0	0	0	4	1	34	0	2
Ayr	2	0	1556	110	10	28	7	0
Dumfries	0	5	0	56	1	0	0	0
Edinburgh	0	58	9	0	1	226	104	0
Inverness	261	35	2	2	0	1	0	0
Perth	1	102	4	23	1	0	5	0
St. Boswells	0	0	20	77	0	0	0	0
Thurso	42	0	2	4	70	1	0	0
